# The Chemistry of HNO: Mechanisms and Reaction Kinetics

**DOI:** 10.3389/fchem.2022.930657

**Published:** 2022-07-05

**Authors:** Radosław Michalski, Renata Smulik-Izydorczyk, Jakub Pięta, Monika Rola, Angelika Artelska, Karolina Pierzchała, Jacek Zielonka, Balaraman Kalyanaraman, Adam Bartłomiej Sikora

**Affiliations:** ^1^ Institute of Applied Radiation Chemistry, Lodz University of Technology, Lodz, Poland; ^2^ Department of Biophysics, Medical College of Wisconsin, Milwaukee, WI, United States

**Keywords:** azanone, nitroxyl, HNO, chemical kinetics, reaction mechanism

## Abstract

Azanone (HNO, also known as nitroxyl) is the protonated form of the product of one-electron reduction of nitric oxide (^•^NO), and an elusive electrophilic reactive nitrogen species of increasing pharmacological significance. Over the past 20 years, the interest in the biological chemistry of HNO has increased significantly due to the numerous beneficial pharmacological effects of its donors. Increased availability of various HNO donors was accompanied by great progress in the understanding of HNO chemistry and chemical biology. This review is focused on the chemistry of HNO, with emphasis on reaction kinetics and mechanisms in aqueous solutions.

## 1 Introduction

The discovery of endogenous enzymatic production of nitric oxide (^•^NO) and its important physiological functions in mammals as a signalling molecule, playing an important role as a vasorelaxant agent, an immune response effector, and a regulator of central nervous system (Ignarro et al., 1987) has established a new paradigm in physiology and medicine (Kerwin et al., 1995). Subsequently, over the past 3 decades, several other small, two or three atomic molecules have been identified as signaling agents. This class of low molecular weight signaling molecules includes ^•^NO, carbon monoxide (CO), hydrogen sulfide (H_2_S) and azanone (HNO) (Wang, 2012; Bianco et al., 2017). Formally, HNO is the protonated product of one-electron reduction of ^•^NO and a highly reactive electrophilic species of growing pharmacological significance (Kemp-Harper et al., 2016; Felker et al., 2019; Fukuto, 2019; Kemp-Harper et al., 2021). Interest in azanone donors and in the biological chemistry of HNO has increased significantly during the last 2 decades, due to the beneficial pharmacological effects of its donors in the treatment of heart failure. Currently, an HNO donor cimlanod (BMS-986231, CXL-1427) is in clinical trials for heart failure treatments (Felker et al., 2019; Felker et al., 2021; Greenberg and Urey, 2021; Lang et al., 2021). This review focuses on the chemistry of aqueous solutions of HNO, with emphasis on the reaction kinetics and mechanisms. The cited HNO rate constants are summarized in Tables 1 and 2.

**TABLE 1:** Second-order rate constants for reactions of HNO with its scavengers.

	*k*_*S*_ [M^−1^s^−1^]	*References*
Inorganic scavengers		
HNO	(8 ± 3) × 10^6^	Shafirovich and Lymar, (2002)
^•^NO	(5.8 ± 0.2) × 10^6^	Lymar et al. (2005)
OH^−^	(4.9 ± 0.5) × 10^4^	Shafirovich and Lymar, (2002)
^3^NO^−^	6.6 × 10^9^	Lymar and Shafirovich, (2007)
O_2_	8 × 10^3^	Liochev and Fridovich, (2003)
	(1.8 ± 0.3) × 10^4^	Smulik et al. (2014)
	3 × 10^3^	Miranda et al. (2003b)
NO_2_^−^	1 × 10^3^	Liochev and Fridovich, (2003)
	(5.0 ± 0.9) × 10^3^	Smulik-Izydorczyk et al. (2017)
NH_2_OH	(4.0 ± 0.3) × 10^3^	Jackson et al. (2009)
	(2.1 ± 0.4) × 10^4^	Smulik-Izydorczyk et al. (2017)
HS^−^	(1.2 ± 0.3) × 10^6^	Smulik et al. (2014)
HSO_3_^−^	(1.2 ± 0.2) × 10^6^	Smulik-Izydorczyk et al. (2017)
S_2_O_3_^2−^	(2.2 ± 0.7) × 10^4^	Smulik-Izydorczyk et al. (2017)
	2.1 × 10^4^	Jackson et al. (2009)
Thiols and selenols		
GSH	2 × 10^6^	Miranda et al. (2003b)
	7.6 × 10^6^	Jackson et al. (2009)
	(3.1 ± 0.6) × 10^6^	Smulik et al. (2014)
NAC	5 × 10^5^	Miranda et al. (2003b)
	(1.4 ± 0.3) × 10^6^	Smulik et al. (2014)
Cys	(4.5 ± 0.9) × 10^6^	Smulik et al. (2014)
Captopril	(6 ± 1) × 10^5^	Smulik et al. (2014)
HSA	(1.4 ± 0.4) × 10^6^	Smulik et al. (2014)
BSA	(1.4 ± 0.3) × 10^6^	Smulik et al. (2014)
Aryl sulfinates		
Benzenesulfinate anions	(4.4 ± 0.9) × 10^4^	Smulik-Izydorczyk et al. (2017)
2-Bromo substituted benzenesulfinate anions (2-BrPhSO_2_^−^)	(5.0 ± 1.2) × 10^4^	Smulik-Izydorczyk et al. (2017)
2-Chloro substituted benzenesulfinate anions (2-ClPhSO_2_^−^)	(3.0 ± 0.7) × 10^4^	Smulik-Izydorczyk et al. (2019)
2-Trifluoromethyl-benzenesulfinates (2-CF_3_PhSO_2_^−^)	(1.1 ± 0.2) × 10^4^	Smulik-Izydorczyk et al. (2019)
*C*- and *S*-nitroso compounds		
Nitrosobenzene	>1.5 × 10^5^	Smulik-Izydorczyk et al. (2017)
2-Nitroso-1-naphthol	(1.0 ± 0.2) × 10^6^	Smulik-Izydorczyk et al. (2017)
S-nitrosoglutathione	(2.4 ± 0.7) × 10^4^	Smulik-Izydorczyk et al. (2017)
Phosphines		
Tris (2,4-dimethyl-5- sulfophenyl)phosphine trisodium salt	9 × 10^5^	Reisz et al. (2011)
Triphenylphosphine-3,3′,3″-trisulfonate	(3.0 ± 0.5) × 10^6^	Smulik-Izydorczyk et al. (2017)
Tris-carboxyethylphosphine	(1.2 ± 0.3) × 10^7^	Smulik-Izydorczyk et al. (2017)
	(8.4 ± 1.8) × 10^6^	Jackson et al. (2009)
C-nucleophiles		
3,4-dimethyl-1-phenyl-pyrazolin-5-one	∼8 × 10^5^	Guthrie et al. (2015)
1,3-Cyclopentanedione (C_5_H_6_O_2_)	(2.8 ± 0.6) × 10^2^	Artelska et al. (2021)
1,3-Cyclohexanedione (C_6_H_8_O_2_)	(2.2 ± 0.4) × 10^3^	Artelska et al. (2021)
1,3-Cycloheptanedione (C_7_H_10_O_2_)	(6.8 ± 1.5) × 10^3^	Artelska et al. (2021)
2-Methyl-1,3-cyclopentanedione (CH_3_C_5_H_6_O_2_)	(3.2 ± 0.9) × 10^3^	Artelska et al. (2021)
2-Methyl-1,3-cyclohexanedione (CH_3_C_6_H_8_O_2_)	(1.1 ± 0.2) × 10^4^	Artelska et al. (2021)
2,4-Piperidinedione	(2.0 ± 0.4) × 10^4^	Artelska et al. (2021)
1-(4-Methoxybenzyl)-2,4-piperidinedione	(1.4 ± 0.3) × 10^4^	Artelska et al. (2021)
2-Thiobarbituric acid	(8.2 ± 1.9) × 10^2^	Artelska et al. (2021)
Meldrum’s acid	(8.7 ± 1.8) × 10^2^	Artelska et al. (2021)
Cyt *c* (Fe^3+^)	2 × 10^4^	Liochev and Fridovich, (2003)
	4 × 10^4^	Miranda et al. (2003b)
Cu,Zn-SOD	8 × 10^4^	Liochev and Fridovich, (2003)
	0.7–1 × 10^6^	Miranda et al. (2003b)
TEMPO	(1.4 ± 0.2) × 10^5^	Samuni et al. (2013)
	6.3 × 10^4^	Jackson et al. (2009)
TEMPOL	(1.4 ± 0.2) × 10^5^	Samuni et al. (2013)
	8 × 10^4^	Miranda et al. (2003b)
3-Carbamoyl-PROXYL	(4.3 ± 0.4) × 10^4^	Samuni et al. (2013)
4-Acetamido-TEMPO	(8 ± 2) × 10^4^	Smulik-Izydorczyk et al. (2017)
TEMPO-9-AC	8 × 10^4^	Cline and Toscano, (2011)
	(9 ± 2) × 10^4^	Smulik-Izydorczyk et al. (2017)
PTIO	(1.4 ± 0.2) × 10^5^	Samuni et al. (2010)
c-PTIO	(1.4 ± 0.2) × 10^5^	Samuni et al. (2010)
	(2.2 ± 0.5) × 10^4^	Bobko et al. (2013), Bobko and Khramtsov (2014)
	(1.2 ± 0.4) × 10^5^	calculated based on the *k*_NH_2_OH_ = 2.1 × 10^4^ M^−1^s^−1^
	(6.8 ± 1.3) × 10^3^	Bobko and Khramtsov, (2015)
	(3.6 ± 1.1) × 10^4^	calculated based on the *k*_NH_2_OH_ = 2.1 × 10^4^ M^−1^s^−1^
Fe(CN)_6_^3−^	(9.6 ± 7.5) × 10^2^	Bobko and Khramtsov, (2015)
	(5.0 ± 4.5) × 10^3^	calculated based on the *k*_NH_2_OH_ = 2.1 × 10^4^ M^−1^s^−1^
NADH, NADPH, ascorbate and other reductants		
NADH	(1.1 ± 0.2) × 10^4^	Jackson et al. (2009)
NADPH	(1.3 ± 0.4) × 10^4^	Jackson et al. (2009)
Ascorbate	1.1 × 10^5^	Jackson et al. (2009)
Trolox	2 × 10^4^	Jackson et al. (2009)
Selenomethionine	9 × 10^3^	Jackson et al. (2009)

**TABLE 2:** Second-order rate constants for reactions of porphyrins with HNO and HNO donors.

Compound	Reactant	Rate constant (M^−1^s^−1^)	References
Mb(Fe^II^)O_2_	HNO	1 × 10^7^	Miranda et al. (2003b)
Mb(Fe^II^)	HNO	>1.4 × 10^4^	Sulc et al. (2004)
Mb(Fe^II^) (origin: horse)	HNO	2.2 × 10^5^	Kumar et al. (2009)
Mb(Fe^II^) (origin: equine)	HNO	3.7 × 10^5^	Zapata et al. (2013)
Hb(Fe^II^) (origin: human)	HNO	∼2.0 × 10^5^	Kumar et al. (2009)
Leghemoglobin lgHb(Fe^II^) (origin: phytoglobin from root nodules of leguminous plants)	HNO	1.2 × 10^5^	Kumar et al. (2009)
Hemoglobin I (cHb(Fe^II^) (origin: invertebrate clam *Lucina pectinate*)	HNO	9.0 × 10^5^	Kumar et al. (2009)
metMb (Mb(Fe^III^))	HNO	8.0 × 10^5^	Miranda et al. (2003b)
	HNO	2.7 × 10^5^ (pH 7.0)	Zapata et al. (2013)
		1.1 × 10^5^ (pH 9.4)	
Catalase(Fe^III^)	HNO	3 × 10^5^	Miranda et al. (2003b)
HRP(Fe^III^)	HNO	2 × 10^6^	Miranda et al. (2003b)
cyt c(Fe^III^)	HNO	4 × 10^4^	Miranda et al. (2003b)
	HNO	2 × 10^4^	Liochev and Fridovich, (2003)
Fe(III) microperoxidase 11 [Fe^III^MP11]^3-^	HNO	(6.4 ± 0.5) × 10^4^ (pH 7)	Suarez et al. (2007)
	HNO	(3.1 ± 0.4) × 10^4^ (pH 10)	Suarez et al. (2007)
[Fe^III^TSPP]^3−^ (Fe(III) *meso*-tetrakis (4-sulfonatophenyl) porphyrinate)	HNO	1 × 10^6^ (pH 7)	Suarez et al. (2007)
	AS	0.5 (pH 7)	Suarez et al. (2007)
[Mn^III^TSPP]^3-^	HNO	∼4 × 10^4^ (pH 7)	Marti et al. (2005)
	HNO	∼9 × 10^4^ (pH 10)	Marti et al. (2005)
[Fe^III^TEPyP]^5+^	AS	(5.4 ± 0.2) × 10^3^ (pH 7)	Suarez et al. (2007)
(Fe(III) Tetrakis N-ethylpyridinium-2-yl porphyne)			
[Fe^III^TEPyP]^5+^	TSHA	(1.1 ± 0.3) × 10^4^ (pH 10)	Suarez et al. (2007)
	Toluene sulfohydroxamic acid		
[Mn^III^TEPyP]^5+^	AS	(1.2 ± 0.1) × 10^4^ (pH 7)	Marti et al. (2005)
	AS	(3.6 ± 0.4) × 10^4^ (pH 10)	Marti et al. (2005)
	TSHA	(8.1 ± 0.3) × 10^1^ (pH 7)	Marti et al. (2005)
	TSHA	(1.00 ± 0.05) × 10^4^ (pH 10)	Marti et al. (2005)
[Mn^III^Br_8_TCPP]^3−^ (Mn(III) β-octa- bromo-*meso*-tetrakis (4-carboxyla-tophenyl)porphyrin)	AS	(3.3 ± 0.3) × 10^3^ (pH 7)	Alvarez et al. (2014)
	TSHA	(4.4 ± 0.5) × 10^3^ (pH 10)	Alvarez et al. (2014)
[Mn^III^Br_8_TSPP]^3−^ (Mn(III) β-octa- bromo-*meso*-tetrakis (4-sulfonato-phenyl)porphyrin)	AS	(3.7 ± 0.3) × 10^3^ (pH 7)	Alvarez et al. (2014)
	TSHA	(3.9 ± 0.2) × 10^3^ (pH 10)	Alvarez et al. (2014)
[Mn^III^T (TriMA)P]^5+^	HNO	(1.1 ± 0.3) × 10^5^ (pH 7)	Alvarez et al. (2014)
(Mn(III) *meso*-tetrakis (4-*N,N,N- trimethylanilinium*) *porphyrin*)			
	AS	(5.4 ± 0.2) × 10^–2^ (pH 7)	Alvarez et al. (2014)
[Mn^III^TCPP]^3−^ (Mn(III) *meso*-tetrakis (4-carboxyla-tophenyl)porphyrin)	HNO	(2.9 ± 0.5) × 10^5^ (pH 7)	Alvarez et al. (2014)
	HNO	(1.6 ± 0.4) × 10^5^ (pH 7)	Alvarez et al. (2014)
[Mn^III^ProtoP]^−^ (Mn(III) protoporphyrin-IX)	HNO	(4.28 ± 0.04) × 10^5^ (pH 7)	Boron et al. (2011)
	HNO	(1.86 ± 0.07) × 10^5^ (pH 10)	Boron et al. (2011)
[Mn^III^HematoP]^−^ (Mn(III) hematoporphyrin IX)	HNO	(1.6 ± 0.3) × 10^5^ (pH 7)	Alvarez et al. (2014)
	HNO	(2.0 ± 0.5) × 10^5^ (pH 10)	Alvarez et al. (2014)
[Mn^III^T-2-PyP]^+^	HNO	(2.1 ± 0.4) × 10^5^ (pH 7)	Alvarez et al. (2014)
(Mn(III) *meso*-tetrakis (2-pirydyl)porphyrin)			
Mb(Mn^III^)	HNO	3.4 × 10^5^ (pH 7.4)	Boron et al. (2011)
(Mn(III) protoporphyrinate IX in apomyoglobin)			

## 2 HNO Chemistry

### 2.1 Nomenclature

Prior to describing HNO chemistry, it is important to briefly discuss the recommended and the used nomenclature. The IUPAC recommended name of HNO is “*azanone*” or “*hydridooxidonitrogen*” (which name is based on additive nomenclature). The commonly used name is “*nitroxyl.*” It has also been used to describe azanone anion, NO^−^ [which IUPAC recommended name is “*oxidonitrate* (*1*−)”]. The term “*nitroxyl*” may be misleading as aminoxyl radicals, of the general formula R_2_N-O^•^, are known as “*nitroxyl radicals.*” Therefore, the reader must be aware that a literature search with the keyword “*nitroxyl*” may yield references to aminoxyl radicals R_2_N-O^•^. In some of the scientific literature names “*nitroso hydrogen*,” “*monomeric hyponitrous acid*,” “*nitrosyl hydride*” or “*hydrogen oxonitrate*,” were also used. For the compliance with the IUPAC recommendations and to avoid ambiguity, we suggest the use of the name “*azanone*” for HNO and “*azanone anion*” for NO^−^.

### 2.2 Structure, Spin States, Acidity

HNO is a simple triatomic molecule and its structure is described by three geometrical parameters: the N-O bond length r_N-O_, the N-H bond length r_N-H_ and the H-N-O angle θ_H-N-O_. The qualitative discussion of the electronic structure of HNO has been given by Orgel (1953) in relation to the properties of the isoelectronic and paramagnetic molecular oxygen and diamagnetic nitroso compounds. He has predicted, that the HNO ground state is singlet. The bent structure of HNO has also been proposed (Orgel, 1953). For the very first time, the molecular geometry of HNO has been experimentally determined by Dalby, based on the constants obtained from the rotational analysis (Dalby, 1958). For the HNO singlet ground state r_N-O_ = 1.212 Å, r_N-H_ = 1.063 Å, and θ_H-N-O_ = 108.6°. HNO structure and NO bond vibrational frequency υ_NO_ has been the subject of numerous theoretical studies [they have been recently reviewed (Zhang, 2013)]. Deprotonation of a singlet ^1^HNO molecule leads to the formation of a NO^−^ anion, which is isoelectronic to molecular oxygen (O_2_) and has a triplet ground state. Due to different spin states of ^1^HNO and ^3^NO^−^, deprotonation of ^1^HNO and the protonation of ^3^NO^−^ in aqueous solution, are spin-forbidden and slow reactions.

The acid-base equilibrium of HNO has been a subject of a long-standing debate. In a pulse radiolysis study on the nitric oxide reduction to NO^−^ a p*K*_a_ value of 4.7 for HNO was reported in 1970 (Grätzel et al., 1970). This value was reevaluated in 2001, based on the theoretical calculations, to 7.2 ± 1.0 (Bartberger et al., 2001). Further theoretical reevaluation led to p*K*_a_(^1^HNO/^3^NO^−^) value of 11.4 (Shafirovich and Lymar, 2002), and 11.6 ± 3.4 (Bartberger et al., 2002). Recently, a p*K*_a_ value of 11.5 was reported, based on the advanced kinetic analysis of Piloty’s acid reaction with aquacobalamin (Polaczek et al., 2021). Based on the agreement between theoretical and experimental values, the use of the value of 11.4 for the p*K*_a_(^1^HNO/^3^NO^−^) is recommended. Shafirovich and Lymar have also estimated the p*K*_a_ values for other HNO acid-base equilibria: p*K*_a_(^1^HNO/^1^NO^−^) ∼ 23, p*K*_a_(^3^HNO/^3^NO^−^) = −1.8 (Shafirovich and Lymar, 2002), suggesting that when formed, ^1^NO^−^ will behave as a strong base, while ^3^HNO will behave as a strong acid.

Electron affinity of ^•^NO, due to its electronic structure, has very low value — *E*_ea_ = 0.026 ± 0.005 eV (Siegel et al., 1972; Travers et al., 1989). ^•^NO reduction to ^1^HNO/^3^NO^−^ in aqueous solutions has long been considered as nonrelevant in biological systems. It is important to note that the reported values of the reduction potential of ^•^NO in aqueous solutions, discussed below, are results of estimation, since this value was not measured directly, due to several experimental limitations, including the instability of HNO. In 2002, it was postulated, that the standard reduction potential for ^•^NO/^3^NO^−^ redox pair is highly negative *E*°(^•^NO/^3^NO^−^) = −0.81 V, and practically outside of the biologically compatible range (Bartberger et al., 2002; Shafirovich and Lymar, 2002). At physiological pH, however, ^•^NO reduction leads to the formation of HNO. Taking into account the postulated p*K*_a_(^1^HNO/^3^NO^−^) value of 11.4, Lymar and co-workers, estimated standard redox potential for ^•^NO/HNO redox pair to be equal to −0.14 V (*E*°(^•^NO/HNO) = −0.14 V, and *E*°’(^•^NO/HNO) = −0.55 V for pH 7) (Shafirovich and Lymar, 2002). Those values have been recently revised to *E*°(^•^NO/HNO) = 0.27 V, and *E*°’(^•^NO/HNO) = −0.14 V for pH 7, based on the computational reevaluation of HNO solvation free energy (Venâncio et al., 2017). Such value, close to the one-electron reduction potential of molecular oxygen (O_2_/O_2_^•–^ redox couple), would suggest the feasibility of ^•^NO reduction to HNO by a variety of cellular enzymatic redox centers. The ^•^NO/HNO interconversion under physiological conditions has been recently discussed in detail (Suarez et al., 2021), and is out of the scope of this article.

### 2.3 Stability of HNO and its Reactivity Towards Small Inorganic Scavengers

It should be noted, that direct experimental determination of the chemical reactivity of HNO is hampered by its rapid dimerization to hyponitrous acid H_2_N_2_O_2_ (Reaction 1a, *k*_1a_ = (8 ± 3) × 10^6^ M^−1^s^−1^) (Shafirovich and Lymar, 2002). This rate constant translates to the half-life time of HNO of ∼0.6 ms, when present at 100 µM concentration in aqueous solution. The acid-base equilibria of H_2_N_2_O_2_ have been reported with p*K*_a_ (H_2_N_2_O_2_/HN_2_O_2_^−^) = 7.0 and p*K*_a_ (HN_2_O_2_^−^/N_2_O_2_^2–^) = 10.9. Therefore, at physiological pH (pH > 7), H_2_N_2_O_2_ exists predominantly in its mono-deprotonated form (HN_2_O_2_^−^). This form is responsible for the instability of H_2_N_2_O_2_ in aqueous solutions and for the formation of N_2_O (Reaction 1b, *k*_1b_ = 5 × 10^–4^ s^−1^, t_1/2_ = 23 min).

$$\text{HNO }+\text{ HNO }\to {\text{ H}}_{2}{\text{N}}_{2}{\text{O}}_{2}$$ (reaction 1a)

$${\text{HN}}_{2}{\text{O}}_{2}^{-}\to {\text{N}}_{2}{\text{O}+\text{HO}}^{-}$$ (reaction 1b)

Due to the instability of HNO in aqueous solutions, in chemical studies on HNO reactivity it is necessary to use HNO donors, that release HNO in a controlled manner, or produce azanone *in situ*, e.g., with the use of radiation techniques (e.g., pulse radiolysis) involving one-electron reduction of ^•^NO by solvated electrons or by hydrogen atoms (H^•^), or photochemical techniques (e.g., laser flash photolysis), including photolysis of alkaline aqueous solutions of trioxodinitrate (N_2_O_3_^2−^, anion of Angeli’s salt). Below, the radiation chemistry of aqueous solutions of ^•^NO and the laser flash photolysis of aqueous solutions of N_2_O_3_^2−^ will be described, as those methods greatly contributed to our understanding of azanone chemistry and to the determination of the rate constants of the reactions involved. Further development of such methodologies for rapid *in situ* formation of HNO/NO^−^and for monitoring their reactions may overcome current obstacles in the studies of HNO/NO^−^chemistry and result in improved knowledge of the azanone chemistry and its biological fates.

#### 2.3.1 Radiolysis of Aqueous Solutions of Nitric Oxide

Early works from the 1960s showed that the radiolysis of aqueous solutions of ^•^NO leads to the formation of nitrite (NO_2_^−^) and N_2_O. The primary transient reactive species produced within approximately 10^–8^ s after the exposition of water to ionizing radiation are hydroxyl radical (^•^OH), hydrated electron (e_aq_^−^), and hydrogen atom (H^•^) [the more detailed description of water radiolysis can be found elsewhere (Bobrowski, 2017)]. The formation of N_2_O and NO_2_^−^ in the irradiated aqueous solutions of nitric oxide is explicable by the mechanism presented below (Reactions 2–8) (Lymar et al., 2005):

eaq-+N•O→N3O- (reaction 2)

H•+N•O→HNO (reaction 3)

O•H + N•O→HONO→NO2-+H+ (reaction 4)

N3O-+N•O→N2O2•- (reaction 5)

H1NO+N•O→N2O2•-+H+ (reaction 6)

$${\text{N}}_{2}{\text{O}}_{2}^{•-}{+}^{•}\text{NO}\to {\text{N}}_{3}{\text{O}}_{3}^{-}$$ (reaction 7)

$${\text{N}}_{3}{\text{O}}_{3}^{-}\to {\text{N}}_{2}{\text{O}+\text{ NO}}_{2}^{-}$$ (reaction 8)

Nitric oxide, being a radical, rapidly reacts with all three primary radical species from water radiolysis (^•^OH, e_aq_^−^ and H^•^). Fast reaction with ^•^OH radical results in the nitrite formation (Reaction 4, *k*_4_ = (1–2) × 10^10^ M^−1^s^−1^) (Treinin and Hayon, 1970; Seddon et al., 1973; Strehlow and Wagner, 1982). Reactions with hydrogen atom (Reaction 3, *k*_3_ = 1.1 × 10^10^ M^−1^s^−1^) (Knight and Sutton, 1967) and hydrated electron (Reaction 2, *k*_2_ = (2.1–3.1) × 10^10^ M^−1^s^−1^) (Gordon et al., 1963; Seddon and Young, 1970; Lymar et al., 2005) lead to ^1^HNO and ^3^NO^−^, respectively. Both, ^1^HNO and its anion ^3^NO^−^ react subsequently with ^•^NO with the formation of hyponitrite radical anion (N_2_O_2_^•−^) (Reactions 5, 6, *k*_5_ = (3.0 ± 0.8) × 10^9^ M^−1^s^−1^, *k*_6_ = (5.8 ± 0.2) × 10^6^ M^−1^s^−1^, p*K*_a_ (HN_2_O_2_^•^) = 5.6 ± 0.3) (Poskrebyshev et al., 2004; Lymar et al., 2005; Poskrebyshev et al., 2008). Further reaction of N_2_O_2_^•−^ with ^•^NO generates N_3_O_3_^−^ anion (Reaction 7, *k*_7_ = (5.4 ± 1.4) × 10^9^ M^−1^s^−1^, p*K*_a_ (HN_3_O_3_) = 3.1), that decomposes to N_2_O and NO_2_^−^ (Reaction 8, *k*_8_ ∼ 3 × 10^2^ s^−1^) (Lymar et al., 2005). The stoichiometric analyses indicate that for 1 mol of N_2_O generated, 2 mol of NO_2_^−^ are produced, and 4 mol of ^•^NO are consumed. Addition of aliphatic alcohols to the solution, leads to scavenging of ^•^OH by the alcohols with the formation of highly reactive ketyl radicals (Reaction 9), capable to reduce ^•^NO to HNO (Reaction 10), thus resulting in an increase in N_2_O yield (Woodward and Sutton, 1966).

O•H+R1R2CHOH→R1R2C•OH+H2O (reaction 9)

N•O+R1R2C•OH→[R1R2C(NO)OH]→R1R2C=O+HNO (reaction 10)

Based on the above described reactions, it can be expected that when HNO is produced by ^•^NO reduction in the presence of the excess of ^•^NO, the transient generation of N_2_O_2_^•−^ and N_3_O_3_^−^ anions will occur resulting in the formation of N_2_O and NO_2_^−^ in 1:1 M ratio, as it was observed in several studies (Suarez et al., 2015; Suarez et al., 2017). It has been shown that N_2_O_2_^•−^ is quite stable both kinetically and thermodynamically with respect to its dissociation to ^•^NO and ^3^NO^−^ (Poskrebyshev et al., 2004). N_2_O_2_^•−^ decay, observed in pulse radiolysis experiments, could be reasonably well described by a second-order kinetics. In the first and rate-determining step, two N_2_O_2_^•−^ molecules disproportionate with the formation of N_2_O_2_^2−^ and generation of two ^•^NO (Reaction 11, 2*k*_11_ = (8.2 ± 0.5) × 10^7^ M^−1^s^−1^).

$${2\text{N}}_{2}{\text{O}}_{2}^{•-}\to {\text{N}}_{2}{\text{O}}_{2}^{2-}{+2}^{•}\text{NO}$$ (reaction 11)

Subsequently, ^•^NO rapidly adds to another N_2_O_2_^•−^ molecule, yielding the N_3_O_3_^−^ anion (Reaction 7), which slowly decomposes to the final products: N_2_O and NO_2_^−^ (Reaction 8) (Poskrebyshev et al., 2008). The standard reduction potentials of N_2_O_2_^•−^ and its protonated form, HN_2_O_2_^•^, were estimated–E°(N_2_O_2_^•−^/N_2_O_2_^2−^) = 0.96 V and E°(HN_2_O_2_^•^, H^+^/H_2_N_2_O_2_) = 1.75 V (Poskrebyshev et al., 2004), suggesting that both species are strong one-electron oxidants.

#### 2.3.2 Photolysis of Trioxodinitrate Solutions

Laser flash photolysis of sodium trioxodinitrate (Angeli’s salt, Na_2_N_2_O_3_) solutions has been used to study the kinetics of ^1^HNO/^3^NO^−^ reactions (Shafirovich and Lymar, 2002, 2003; Lymar et al., 2005). Angeli’s salt anion in strongly alkaline solutions exists exclusively in fully deprotonated form (N_2_O_3_^2−^) and is stable for hours. It has been shown, that steady state UV photolysis of alkaline Angeli’s salt solutions saturated with N_2_ results in the formation of nitrite and N_2_O, whereas the photolysis of solutions equilibrated with air, or saturated with oxygen, leads to the formation of ONOO^−^ (Donald et al., 1986; Shafirovich and Lymar, 2002). The kinetics of ONOO^−^ formation has been studied with the use of laser flash photolysis technique. It has been determined that the rate of ONOO^−^ formation at fixed pH is independent of oxygen concentration and it has been proposed, that the rate-determining step is the ^1^HNO deprotonation in the reaction with OH^−^. The proposed mechanism assumes that ONOO^−^is formed only *via* the reaction between ^3^NO^−^and O_2_, and can be described by reaction sequence presented below (Reactions 12–15). The first step is the heterolytic photochemical cleavage of N_2_O_3_^2−^, resulting in the formation of NO^−^ in its singlet state and NO_2_^−^ (Reaction 12). Azanone anion in its singlet state is an extremely strong base (p*K*_a_ (^1^HNO/^1^NO^−^) ∼ 23) and is protonated by water instantaneously (Reaction 13). The next and rate-determining step is deprotonation of ^1^HNO to ^3^NO^−^ (p*K*_a_ (^1^HNO/^3^NO^−^) ∼ 11.4) (Reaction 14). The process of ONOO^−^ generation is completed by a rapid and spin-allowed reaction of ^3^NO^−^ and ^3^O_2_ (Reaction 15). The results obtained in those experiments were used to determine the second order rate constants of (Reaction 14 and Reaction 1a) (*k*_14_ = (4.9 ± 0.5) × 10^4^ M^−1^s^−1^ and *k*_1a_ = (8 ± 3) × 10^6^ M^−1^s^−1^) (Shafirovich and Lymar, 2002).

$${\text{N}}_{2}{\text{O}}_{3}^{2-}\left(+\text{h}\upsilon \right)\to^{1}{\text{NO}}^{-}{+\text{NO}}_{2}^{-}$$ (reaction 12)

NO-1+H2O→H1NO+OH- (reaction 13)

H1NO+OH-→NO-3+H2O (reaction 14)

N3O-+O23→ONOO- (reaction 15)

The second order rate constant of ^3^NO^−^ reaction with ^3^O_2_ (Reaction 15) was estimated to be equal to (2.7 ± 0.2) × 10^9^ M^−1^s^−1^ (Shafirovich and Lymar, 2002). The photolysis of Angeli’s salt solutions at neutral pH, where HN_2_O_3_^−^ is the main acid-base form, also resulted in the formation of ONOO^−^. The estimated yield of ONOO^−^ formation at neutral pH was close to 0.25, and based on that observation it was proposed, that photolysis of aqueous solutions of HN_2_O_3_^−^ leads to the formation of ^3^HNO and ^1^HNO in a 1:3 ratio (Reactions 16, 17). As mentioned earlier, ^3^HNO is a strong acid (p*K*_a_ (^3^HNO/^3^NO^−^) = −1.8), and should dissociate rapidly to ^3^NO^−^ in buffered solutions (Reaction 18) followed by rapid reaction with O_2_ (Reaction 15).

HN2O2-(+hυ)→H3NO (reaction 16)

HN2O2-(+hυ)→H1NO (reaction 17)

H3NO+H2O→NO-3+H3O+ (reaction 18)

The distribution of the primary photolysis products of Angeli’s salt aqueous solution at neutral pH (22% ^3^NO^−^, 58% ^1^HNO, 20% ^•^NO), was determined in a follow-up study (Lymar and Shafirovich, 2007). It has also been determined that the photolysis of aqueous solutions of amine-based diazeniumdiolates results in the generation of ^•^NO (the distribution of the primary photolysis products of DTPA NONOate: 3% ^3^NO^−^, 12% ^1^HNO, 85% ^•^NO). Laser flash photolysis experiments also allowed the determination of the second-order rate constant of the ^1^HNO reaction with ^3^NO^−^ (Reaction 19, *k*_19_ = 6.6 × 10^9^ M^−1^s^−1^) (Lymar and Shafirovich, 2007).

H1NO+NO-3→HN2O2- (reaction 19)

#### 2.3.3 Reaction of Azanone With Molecular Oxygen

One of the most intriguing aspects of ^1^HNO chemistry is its reactivity toward molecular oxygen. As discussed above, the rapid reaction between triplet azanone anion (^3^NO^−^) and O_2_ leads to the formation of a strong biological oxidizing and nitrating agent, ONOO^−^ (Reaction 15, *k*_15_ = (2.7 ± 0.2) × 10^9^ M^−1^s^−1^) (Shafirovich and Lymar, 2002). The formation of ONOO^−^, as a transient product, was postulated in the oxidation reaction of potassium hydroxylamine-*N*-sulfonate (KO_3_SNHOH) (Belt and Baenziger, 1957) in oxygenated alkaline aqueous solutions (Ackermann and Powell, 1967). It has been proposed earlier, that this compound is an HNO donor (Ackermann and Powell, 1966). Formation of ONOO^−^ was also confirmed for decomposition of Angeli’s salt or 2-bromo derivative of Piloty’s acid in alkaline solutions (Yagil and Anbar, 1964; Kirsch and de Groot, 2002; Smulik-Izydorczyk et al., 2019). ONOO^−^ was also shown to be formed in oxygenated aqueous alkaline solutions of hydroxylamine, with azanone anion NO^−^ proposed as a transient product reacting with O_2_ to form ONOO^−^ (Yagil and Anbar, 1964; Hughes and Nicklin, 1971; Seel and Kaschuba, 1975).

In the case of ^1^HNO, the product of its reaction with O_2_ for many years has not been fully identified. It has been shown, that during the decomposition of Angeli’s salt in aerated aqueous solutions at neutral pH an oxidant is formed in the reaction of HNO with O_2_, capable of oxidizing dihydrorhodamine 123 (DHR) or dichlorodihydrofluorescein (DCFH) to their fluorescent products, rhodamine 123 (Rh) and dichlorofluorescein (DCF), respectively (Liochev and Fridovich, 2001; Miranda et al., 2001; Kirsch and de Groot, 2002). The yield of Rh was similar for both oxidants–Angeli’s salt and authentic ONOO^−^. Similar was also the effect of CO_2_, known to increase the yield of peroxynitrite-derived oxidation products (Miranda et al., 2001). It has also been shown, that the yield of DHR and NADH oxidation was similar for Angeli’s salt and SIN-1, a slow donor of ONOO^−^ under aerobic conditions *via* co-generation of superoxide radical anion (O_2_^•−^) and ^•^NO (Kirsch and de Groot, 2002). The yield of nitrate was also similar in the case of aerated aqueous solutions of Angeli’s salt and SIN-1 (Kirsch and de Groot, 2002). All those observations suggested that ONOO^−^ is the product of the reaction of ^1^HNO with O_2_ (Liochev and Fridovich, 2001; Miranda et al., 2001; Kirsch and de Groot, 2002). There were, however, also some significant differences in the effects observed with Angeli’s salt and authentic ONOO^−^. It has been shown that Angeli’s salt, unlike ONOO^−^, is unable to oxidize phenols to their dimeric fluorescent products. This observation led to the conclusion that ONOO^−^ is not formed in the reaction of HNO with O_2_ (Miranda et al., 2001; Miranda et al., 2002). As a result, the possibility of ONOO^−^ formation from the reaction between ^1^HNO and O_2_ has been a matter of a long-term controversy. This was, in part, due to the lack of molecular probes that unambiguously identify and quantify ONOO^−^under the studied conditions.

The development of boronate-based probes for ONOO^−^ provided an opportunity for quantitative measurement of ONOO^−^ formation at neutral pH. Boronic acids and their esters react directly and rapidly with ONOO^−^ (k ∼ 10^5^–10^6^ M^−1^s^−1^), with the formation of corresponding phenols as major products (Sikora et al., 2009; Zielonka et al., 2010; Zielonka et al., 2012; Sikora et al., 2013; Dębowska et al., 2016; Rios et al., 2016). It has been shown, that the reaction of boronates with ONOO^−^ yields unique minor peroxynitrite-specific products (Sikora et al., 2009; Sikora et al., 2011; Sikora et al., 2013; Zielonka et al., 2015; Zielonka et al., 2016b; Zielonka et al., 2021). It has been proposed, that the reaction between ONOO^−^ and boronic acid leads to the formation of ONOO^−^ adduct, followed by the heterolytic (major pathway) or homolytic (minor pathway) O-O bond cleavage, resulting in the formation of phenols (major pathway) and minor radical-derived products (Sikora et al., 2011). It has been proposed, that the formation of the products of both major and minor pathways in a specific ratio can be used as a “peroxynitrite fingerprint” (Sikora et al., 2011; Zielonka et al., 2015; Zielonka et al., 2016a; Szala et al., 2020; Grzelakowska et al., 2021; Zielonka et al., 2021; Grzelakowska et al., 2022). This strategy has been applied to unambiguously determine the identity of the product of HNO reaction with O_2_ (Smulik et al., 2014). The formation of ONOO^−^ in the reaction of HNO with O_2_ in aqueous solutions at neutral pH was confirmed based on the oxidation of the boronate probes in the solutions of Angeli’s salt, and the formation of ONOO^−^-specific products (Smulik et al., 2014). The second order rate constant for ^1^HNO reaction with O_2_ at pH 7.4 (Reaction 20) (*k*_20_ = (1.8 ± 0.3) × 10^4^ M^−1^s^−1^) has been determined with the use of a set of HNO scavengers of known reactivity and the competition kinetics approach (Smulik et al., 2014). It should be noted that this value is significantly higher than previously reported 8 × 10^3^ M^−1^s^−1^ (Liochev and Fridovich, 2003) or 3 × 10^3^ M^−1^s^−1^ (Miranda et al., 2003b) - see Table 1.

H1NO+O2→ONOO-+H+ (reaction 20)

#### 2.3.4 Reaction of Azanone With Nitrite

The estimation of *k*_20_ reported by Liochev and Fridovich was based on the effect of NO_2_^−^ on the aerobic decomposition of Angeli’s salt. The decomposition of Angeli’s salt anion in aqueous solutions at neutral pH leads to the formation of HNO and NO_2_^−^ (Reaction 21). Due to the occurrence of the opposite reaction – HNO scavenging by NO_2_^−^ (Reaction 22), the observed rate of Angeli’s salt decomposition is significantly slower in solutions containing millimolar concentrations of NO_2_^−^. Based on the observed effect of NO_2_^−^ on the Angeli’s salt decomposition in aerated solutions, the second-order rate constant of HNO reaction with NO_2_^−^ (Reaction 22) was estimated to be equal to 1 × 10^3^ M^−1^s^−1^ (Liochev and Fridovich, 2003).

HN2O3-→NO2-+ H1NO (reaction 21)

NO2-+H1NO→HN2O3- (reaction 22)

**SCHEME 1:**
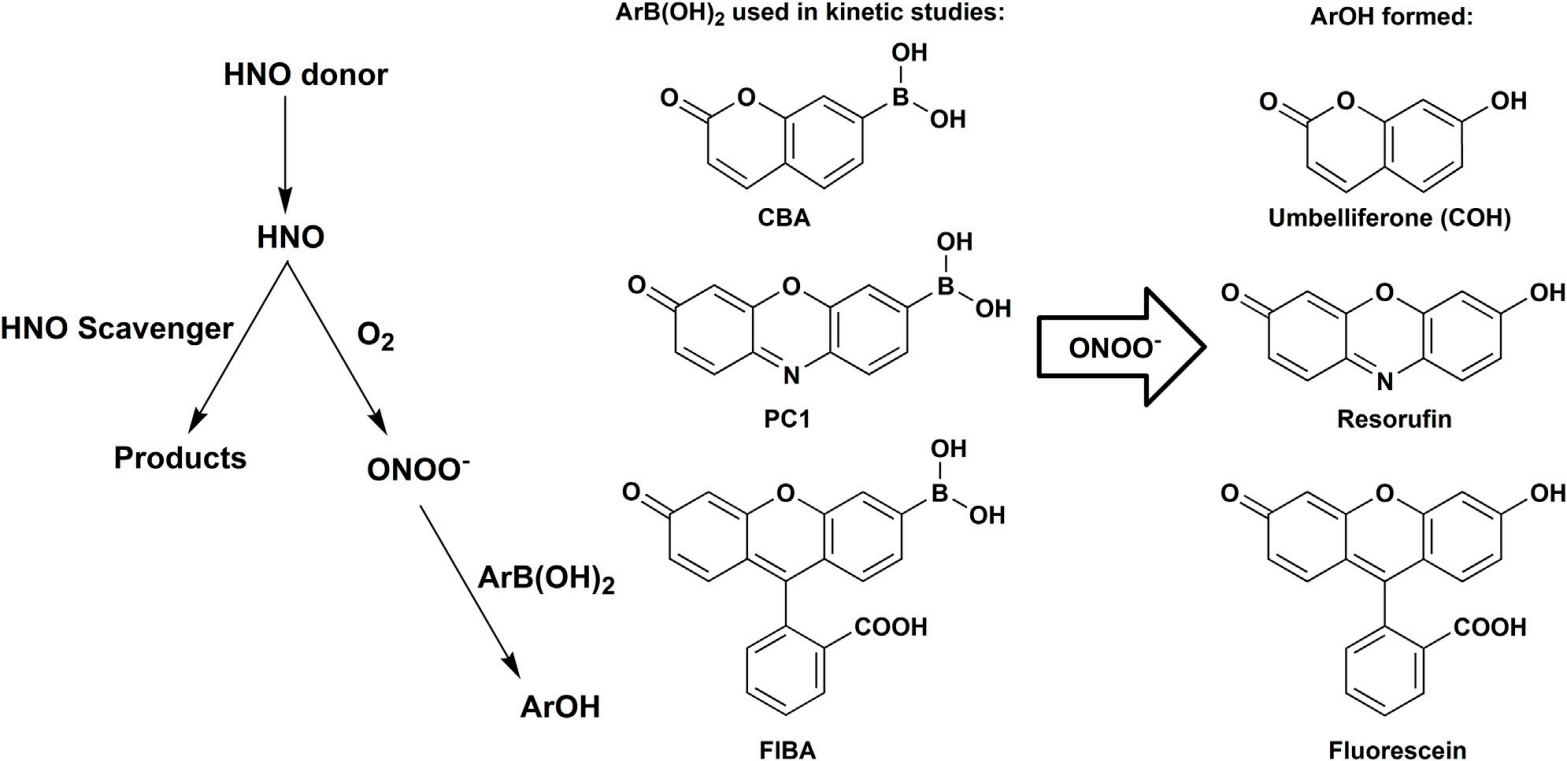
Reaction model used to determine the rate constants of the reactions between HNO and its scavengers with the use of boronate probes, the competition kinetic approach and HNO reaction with O_2_ as a reference reaction.

The use of boronate fluorogenic/colorimetric probes, in combination with a competition kinetics approach was proposed as a novel approach to study the kinetics of HNO reactions (Smulik et al., 2014). Over the last 7 years, the usefulness of this methodology was demonstrated in the studies of HNO reactivity toward a number of its scavengers (Smulik et al., 2014; Smulik-Izydorczyk et al., 2017; Smulik-Izydorczyk et al., 2019; Artelska et al., 2021; Smulik-Izydorczyk et al., 2021). The principle of this method is based on the competition between O_2_ and the scavenger of interest for their reaction with HNO released from Angeli’s salt or other HNO donor. Reaction of HNO with O_2_ results in the formation of ONOO^−^ (Reaction 20), which oxidizes boronate probe (ArB(OH)_2_, e.g., coumarin 7-boronic acid (CBA), resorufin boronate derivative (PC1), fluorescein boronate derivative (FlBA)—see Scheme 1) to the corresponding phenolic product (ArOH, e.g., umbelliferone, resorufin or fluorescein), that can be easily monitored by UV-Vis absorption or fluorescence spectroscopy.

The rate of accumulation of the phenolic reporter (ArOH) over time can be expressed by Eq. 1,

$$v=\frac{d\left[\mathrm{ArOH}\right]}{\mathrm{dt}}=k_{\mathrm{ArB}{\left(\mathrm{OH}\right)}_{2}\;}\left[\mathrm{ArB}{\left(\mathrm{OH}\right)}_{2}\right]\left[\mathrm{ONO}O^{-}\right]$$ (1)

where $k_{\text{ArB}{\left(\text{OH}\right)}_{2}}$ is the rate constant for the boronate probe reaction with ONOO^−^ ( $k_{\text{ArB}{\left(\text{OH}\right)}_{2}}$ ∼ 10^6^ M^−1^s^−1^). Changes in the concentrations of HNO and ONOO^−^ over time are expressed by Eqs 2, 3.

$$\frac{d\left[\mathrm{HNO}\right]}{\mathrm{dt}}=k_{\mathrm{donor}}\left[\mathrm{donor}\right]-k_{\mathrm{scavenger}}\left[\mathrm{scavenger}\right]\left[\mathrm{HNO}\right]-k_{\mathrm{oxygen}}\left[O_{2}\right]\left[\mathrm{HNO}\right]$$ (2)

$$\frac{d\left[\mathrm{ONO}O^{-}\right]}{\mathrm{dt}}=k_{\mathrm{oxygen}}\left[O_{2}\right]\left[\mathrm{HNO}\right]-k_{\mathrm{ArB}{\left(\mathrm{OH}\right)}_{2}}\left[\mathrm{ArB}{\left(\mathrm{OH}\right)}_{2}\right]\left[\mathrm{ONO}O^{-}\right]$$ (3)

where *k*_donor_ is the rate constant of HNO donor decomposition, and *k*_scavenger_ and *k*_oxygen_ are the rate constants of azanone reaction with its scavenger and O_2_, respectively. To solve Eqs 2, 3, a steady state approximation was made (Eqs 4, 5).

$$\frac{d\left[\mathrm{HNO}\right]}{\mathrm{dt}}=0$$ (4)

$$\frac{d\left[\mathrm{ONO}O^{-}\right]}{\mathrm{dt}}=0$$ (5)

The solution leads to Eqs 6, 7.

$$\left[\mathrm{HNO}\right]=\frac{k_{\mathrm{donor}}\left[\mathrm{donor}\right]}{k_{\mathrm{scavenger}}\left[\mathrm{scavenger}\right]+k_{\mathrm{oxygen}}\left[O_{2}\right]}$$ (6)

$$\left[\mathrm{ONO}O^{-}\right]=\frac{k_{\mathrm{oxygen}}\left[O_{2}\right]}{k_{\mathrm{ArB}{\left(\mathrm{OH}\right)}_{2}\;}\left[\mathrm{ArB}{\left(\mathrm{OH}\right)}_{2}\right]}\left[\mathrm{HNO}\right]=\frac{k_{\mathrm{oxygen}}\left[O_{2}\right]}{k_{\mathrm{ArB}{\left(\mathrm{OH}\right)}_{2}\;}\left[\mathrm{ArB}{\left(\mathrm{OH}\right)}_{2}\right]}\cdot \frac{k_{\mathrm{donor}}\left[\mathrm{donor}\right]}{k_{\mathrm{scavenger}}\left[\mathrm{scavenger}\right]+k_{\mathrm{oxygen}}\left[O_{2}\right]}$$ (7)

The rate of ArOH formation *v*_i_ in the presence of azanone scavenger is expressed by Eq. 8, whereas, in the absence of that scavenger, it is expressed by Eq. 9.

$$v_{i}=k_{\mathrm{donor}}\left[\mathrm{donor}\right]\cdot \frac{k_{\mathrm{oxygen}}\left[O_{2}\right]}{k_{\mathrm{scavenger}}\left[\mathrm{scavenger}\right]+k_{\mathrm{oxygen}}\left[O_{2}\right]}$$ (8)

$$v_{0}=k_{\mathrm{donor}}\left[\mathrm{donor}\right]$$ (9)

Comparison of Eqs 8, 9 results in Eq. 10 describing the competition for HNO between O_2_ and the scavenger studied.

$$\frac{v_{0}}{v_{i}}=1+\frac{k_{\mathrm{scavenger}}}{k_{\mathrm{oxygen}}}\cdot \frac{\left[\mathrm{scavenger}\right]}{\left[O_{2}\right]}$$ (10)

It should be noted that this model is based on several assumptions, including: 1) the HNO scavenger of interest does not affect the rate of HNO release from the donor; 2) the scavenger does not react with ONOO^−^, or such reaction is outcompeted by the reaction between ONOO^−^and the boronate probe; 3) the scavenger does not react with or interferes with the detection of the product of oxidation of the boronic probe; and 4) the amount of HNO released is significantly lower than the amount of the scavenger and of O_2_ in the solution.

With the use of this approach and a boronate derivative of resorufin, the rate constant of the reaction of HNO with NO_2_^−^ was determined to be equal to (5.0 ± 0.9) × 10^3^ M^−1^s^−1^ (Smulik-Izydorczyk et al., 2017), 5-fold higher than reported previously (Liochev and Fridovich, 2003).

#### 2.3.5 Reaction of Azanone With Hydroxylamine

It has been shown, that the reaction of azanone with hydroxylamine (NH_2_OH) results in the formation of molecular nitrogen (N_2_) and water (Reaction 23) (Bonner et al., 1978). Jackson et al. (2009) estimated the rate constant for this reaction as *k*_23_ = (4.0 ± 0.3) × 10^3^ M^−1^s^−1^. Recent analysis of the competition between O_2_ and NH_2_OH for HNO using the boronate reporter for ONOO^−^, resulted in the rate constant equal to (2.1 ± 0.4) × 10^4^ M^−1^s^−1^ (Smulik-Izydorczyk et al., 2017).

$${\text{HNO}+\text{NH}}_{2}\text{OH}\to \to {\text{N}}_{2}{+2\text{H}}_{2}\text{O}$$ (reaction 23)

#### 2.3.6 Reaction of Azanone With HS^−^, Thiosulfate, and Sulfite

Published theoretical study predicted that HNO does not react with hard nucleophiles – water or alcohols (Bartberger et al., 2001). However, experimental data show, that it is highly reactive, towards soft nucleophiles. Using the above-described competition kinetics approach, it was shown that HNO reacts with HS^−^, HSO_3_^−^ and S_2_O_3_^2−^ anions (Smulik et al., 2014; Smulik-Izydorczyk et al., 2017). The products of those reactions were not identified, but the corresponding adducts can be expected as primary reaction products (Reactions 24–26).

$${\text{HNO}+\text{HS}}^{-}{+\text{H}}^{+}\to \text{HSNHOH}$$ (reaction 24)

HNO+HSO3-+H+→OSO2-NHOH (reaction 25)

$${\text{HNO}+\text{S}}_{2}{\text{O}}_{3}^{2-}{+\text{H}}^{+}\to^{-}{\text{O}}_{3}\text{SSNHOH}$$ (reaction 26)

$${\text{HSNHOH}+\text{HS}}^{-}\to {\text{HS}}_{2}^{-}{+\text{NH}}_{2}\text{OH}$$ (reaction 27)

O3-SSNHOH+S2O32-+H+→S4O62-+NH2OH (reaction 28)

**SCHEME 2:**
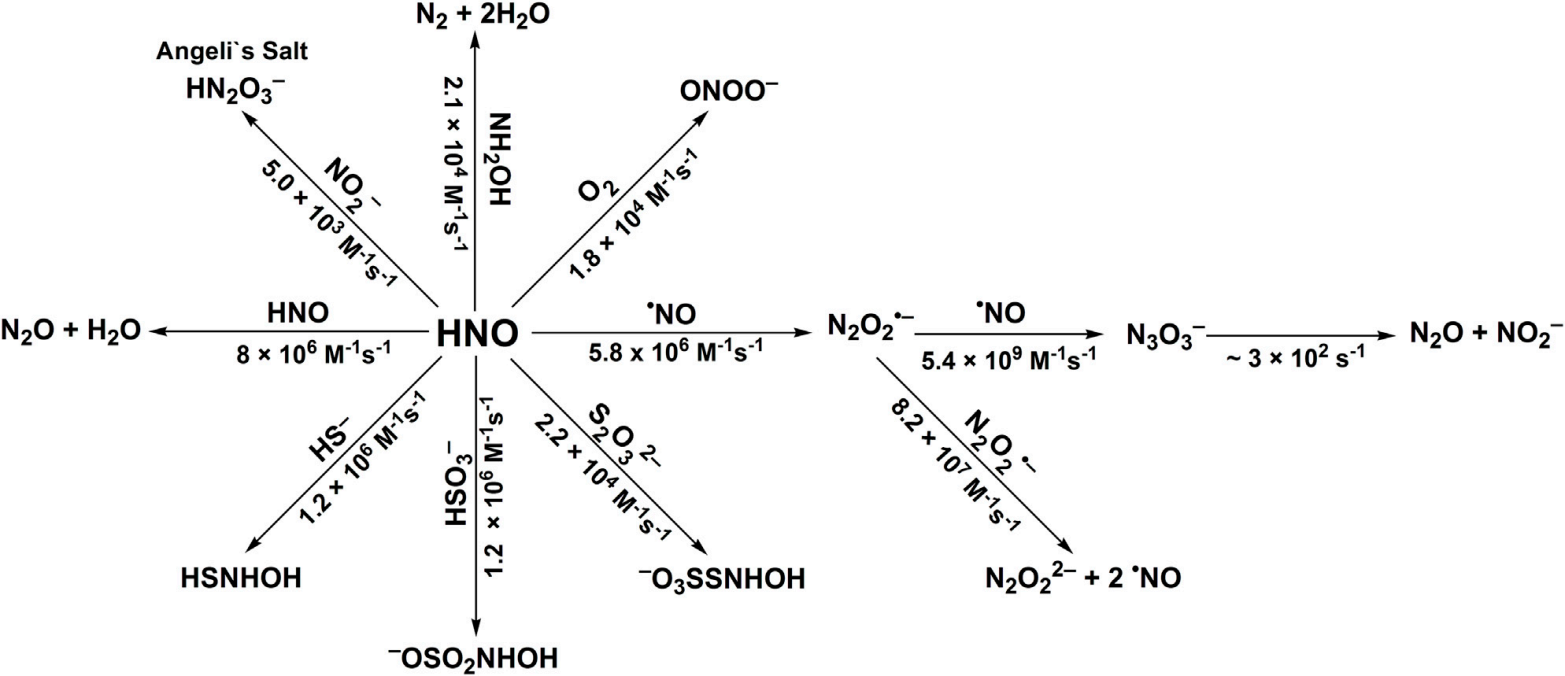
Reactivity of azanone towards selected inorganic scavengers.

The second-order rate constants were determined to be equal to: *k*_24_ = (1.2 ± 0.3) × 10^6^ M^−1^s^−1^, *k*_25_ = (1.2 ± 0.2) × 10^6^ M^−1^s^−1^ and *k*_26_ = (2.2 ± 0.7) × 10^4^ M^−1^s^−1^, for HS^−^, HSO_3_^−^, and S_2_O_3_^2−^, respectively (Smulik et al., 2014; Smulik-Izydorczyk et al., 2017). The rate constant for HNO reaction with S_2_O_3_^2−^ (Reaction 26) has also been reported by Jackson to be equal to 2.1 × 10^4^ M^−1^s^−1^ (Jackson et al., 2009) in perfect agreement with the competition method described above. In case of the reaction between HS^−^ and HNO (Reaction 24) the product formed, HSNHOH, may react with HS^−^ to form HS_2_^−^ and release NH_2_OH (Reaction 27). Similarly, the reaction of ^−^O_3_SSNHOH adduct (the product of Reaction 26) with S_2_O_3_^2−^ could result in the formation of NH_2_OH and S_4_O_6_^2−^ anion (Reaction 28). The stoichiometric analyses of such reaction should also take into account the possibility of the reaction between HNO and released NH_2_OH (Reaction 23). The recent study of Zarenkiewicz et al. (2021) on HNO reaction with H_2_S shows that this reaction leads to the formation of hydrogen polysulfides (H_2_S_n_) or sulfur S_8_, depending on the relative concentrations of H_2_S and HNO. The reactivity of HNO towards selected inorganic scavengers is summarized in Scheme 2 and Table 1.

### 2.4 Reactivity of HNO Towards Organic Nucleophiles

#### 2.4.1 Reaction of Azanone With Thiols, Selenols and Hydropersulfides

One of the earliest reports describing the oxidation of thiols in solutions of HNO donors was by Doyle et al. (1988). The authors have shown that in Angeli’s salt solution in 40% aqueous acetonitrile at pH 7.8, thiophenol is oxidized to phenyl disulfide, with concomitant formation of NO_2_^−^ and NH_2_OH (Reaction 29).

$${2\text{C}}_{6}{\text{H}}_{5}{\text{SH}+\text{HN}}_{2}{\text{O}}_{3}^{-}\to \to {\left({\text{C}}_{6}{\text{H}}_{5}\text{S}\right)}_{2}{+\text{NO}}_{2}^{-}{+\text{NH}}_{2}\text{OH}$$ (reaction 29)

The proposed reaction mechanism involves HNO generation and its subsequent reaction with thiophenol resulting in the formation of C_6_H_5_SNHOH as a transient product (Reaction 30). The reaction of latter with C_6_H_5_SH was proposed to explain the formation of disulfide and NH_2_OH (Reaction 31). NO_2_^−^ was the byproduct of the decomposition of Angeli’s salt (Reaction 21).

$${\text{C}}_{6}{\text{H}}_{5}\text{SH}+\text{HNO}\to {\text{C}}_{6}{\text{H}}_{5}\text{SNHOH}$$ (reaction 30)

$${\text{C}}_{6}{\text{H}}_{5}{\text{SNHOH}+\text{C}}_{6}{\text{H}}_{5}\text{SH}\to {\left({\text{C}}_{6}{\text{H}}_{5}\text{S}\right)}_{2}{+\text{NH}}_{2}\text{OH}$$ (reaction 31)

Subsequently, it was shown that high concentrations of L-cysteine inhibit completely the vasorelaxant response of isolated rat aortic rings to HNO generated from Angeli’s salt (Pino and Feelisch, 1994). Based on the known reaction of thiols with nitrosoarenes (Kazanis and McClelland, 1992) (Reactions 32, 33), it has been proposed and shown, that HNO reacts with thiols to form *N*-hydroxysulfenamides (Reaction 34), that can rearrange to generate a sulfinamides (Reaction 35), as stable products (Shoeman and Nagasawa, 1998; Wong et al., 1998; Shen and English, 2005).

RSH+ArNO→RSN(Ar)OH (reaction 32)

RSN(Ar)OH→RS(O)NHAr (reaction 33)

RSH+HNO→RSNHOH (reaction 34)

$$\text{RSNHOH}\to \text{RS}\left(\text{O}\right){\text{NH}}_{2}$$ (reaction 35)

To the best of our knowledge, due to an apparent instability of thiols *N*-hydroxysulfenamides, all attempts to chemically characterize those primary products of HNO reaction with thiols were thus far unsuccessful.

**SCHEME 3:**
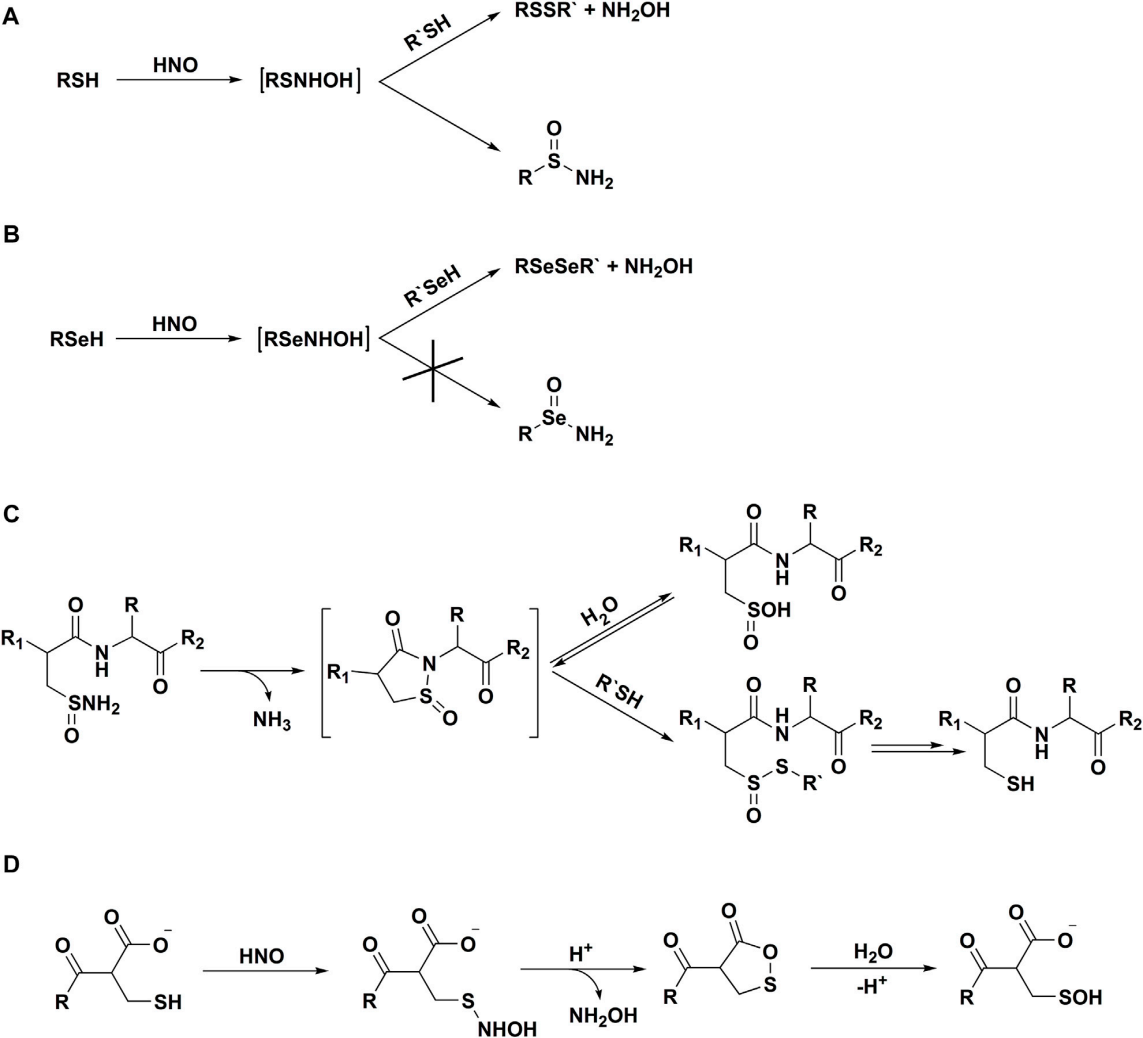
Reactivity of azanone towards thiols and selenols. Reaction of HNO with thiols **(A)** and selenols **(B)**; **(C)** chemical transformations of sulfinamides, **(D)** reaction of HNO with C-terminal cysteines.

In 2016 Bianco et al. (2016) reported, that selenols are also highly reactive towards HNO and demonstrated that reactions of organoselenols and HNO result only in diselenide formation. It has been proposed, that such product’s profile is unique to selenols and selenoproteins (Scheme 3B).

Exposure of reduced glutathione (GSH) to HNO was shown to lead to the formation of both the sulfinamide derivative (GS(O)NH_2_) and the glutathione disulfide (oxidized glutathione, GSSG) (Donzelli et al., 2006). Higher GSH concentrations favored a higher [GSSG]/[GS(O)NH_2_] ratio, suggesting that both GSSG and GS(O)NH_2_ are formed from a single transient intermediate, supporting the hypothesis of GSNHOH formation (Scheme 3A). It has also been shown, that formation of GS(O)NH_2_ is not observed with other reactive nitrogen species (RNS): ^•^NO, nitrogen dioxide (^•^NO_2_), ONOO^−^, or N_2_O_3_, making GS(O)NH_2_ a unique and HNO-specific product. GS(O)NH_2_ was, therefore, used as a marker for HNO generation from several biologically-relevant pathways: thiol-mediated decomposition of *S*-nitrosothiols, peroxidase-dependent NH_2_OH and *N*-hydroxy-L-arginine oxidation (Donzelli et al., 2006).

It has been long assumed that HNO-driven oxidative transformation of thiols to sulfinamides is irreversible. Keceli and Toscano (2012) have examined the stability of HNO-derived sulfinamides in several systems, including small organic molecules, peptides, and a protein and observed that sulfinamides can be reduced back to the corresponding thiols in the presence of thiol excess. It was also shown that upon prolonged incubation of sulfinamides in water, sulfinic acid (RS(O)OH) is formed. However, in the presence of thiols, the reduction of sulfinamides is effectively outcompeting the hydrolysis process. The proposed mechanism of peptide sulfinamide reduction involving a cyclic transient product is presented in Scheme 3C.

The same authors have also shown that in the case of *C*-terminal cysteines the nature of HNO-derived modifications is highly affected by the *C*-terminal carboxylate (Keceli and Toscano, 2014). In this case, the formation of sulfinamides is not observed. It was proposed, that the *N*-hydroxysulfenamide formed in HNO reaction with the thiol undergoes intramolecular cyclization to the transient cyclic product, that is hydrolyzed to sulfenic acid (RSOH) (Scheme 3D).

Several groups studied the kinetics of the reaction of HNO with GSH. Based on the effect of GSH on the rate of Angeli’s salt decomposition in aqueous solution at pH 7.4 in the presence of NO_2_^−^ and O_2_, it was concluded that the rate constant for the reaction of GSH with NO^−^/HNO is significantly higher than 1 × 10^5^ M^−1^s^−1^ (Liochev and Fridovich, 2003). In an independent study, the second order rate constants for the reactions of HNO with GSH and *N*-acetyl cysteine (NAC) were determined to be equal to 2 × 10^6^ M^−1^s^−1^ and 5 × 10^5^ M^−1^s^−1^, respectively (Miranda et al., 2003b). The rate constant for HNO reaction with GSH has also been reported to be equal to 7.6 × 10^6^ M^−1^s^−1^ (Jackson et al., 2009). More recent reexamination of the reactivity of selected thiols, based on their competition with O_2_ for HNO (using boronate probes for ONOO^−^), yielded the following values of the rate constants in aqueous solutions at pH 7.4: cysteine (*k*_Cys_ = (4.5 ± 0.9) × 10^6^ M^−1^s^−1^, p*K*_a_ = 8.3), glutathione (*k*_GSH_ = (3.1 ± 0.6) × 10^6^ M^−1^s^−1^, p*K*_a_ = 8.8), *N*-acetylcysteine (*k*_NAC_ = (1.4 ± 0.3) × 10^6^ M^−1^s^−1^, p*K*_a_ = 9.5) and captopril (*k*_Cap_ = (6 ± 1) × 10^5^ M^−1^s^−1^, p*K*_a_ = 9.8) (Smulik et al., 2014). It was shown, that for those small molecular weight thiols, there is an inverse correlation between the determined rate constants and p*K*_a_ values of SH groups: the higher was the value of thiol’s p*K*_a_, the lower was the reaction rate constant. This strongly suggests that the deprotonated SH group (i.e., thiolate anion) is the active form reacting with HNO. The second order rate constants for HNO reaction with protein-bound thiols were also determined in that study for human and bovine serum albumins (p*K*_a_ ∼ 8.5 for Cys34) (*k*_HSA_ = (1.4 ± 0.4) × 10^6^ M^−1^s^−1^, *k*_BSA_ = (1.4 ± 0.3) × 10^6^ M^−1^s^−1^). Such a high reactivity of thiols towards HNO, together with high concentrations of GSH and albumin in cells and plasma, respectively, suggest that thiols may be the potential targets of HNO in biological systems.

The pH-dependent kinetics of HNO reactions with selected thiols–glutathione, *N*-acetylcysteine, bovine serum albumin and human serum albumin was also recently reported (Smulik-Izydorczyk et al., 2021). It was shown, that the observed rate constant (*k*_obs_) can be expressed as a function of pH, which depends on thiol p*K*_a_, and the rate constants of azanone reaction with RS^−^ (*k*_thiolate_) and with RSH (*k*_thiol_) (Eq. 11):

$$k_{\mathrm{obs}}=\frac{k_{\mathrm{thiolate}}\cdot 10^{-pK_{a}}\;+\;k_{\mathrm{thiol}}\cdot 10^{-\mathrm{pH}}}{10^{-pK_{a}}\;+\;10^{-\mathrm{pH}}}$$ (11)

The results of that study showed, that the reaction of HNO with thiolate (RS^−^) is favored (*k*_thiolate_ ∼ 10^7^ M^−1^s^−1^) over reactions with the thiol (RSH, *k*_thiol_ = 10^5^–10^6^ M^−1^s^−1^). Such observation is consistent with the observed inverse correlation between the reaction rate constant and the p*K*_a_ value of the thiol.

Another class of thiol-related, biologically important sulfur nucleophiles are hydropersulfides (RSSH) (Saund et al., 2015; Fukuto and Hobbs, 2021). At physiological pH hydropersulfides (RSSH) are significantly more nucleophilic than thiols (RSH), due to their lower p*K*_a_ [e.g., p*K*_a_ (GSSH) = 5.45 ± 0.03, 3.5 units below the p*K*_a_ of GSH (Benchoam et al., 2020)] and increased nucleophilicity of their anions RSS^−^. One may expect, that RSSH/RSS^−^ are highly reactive towards HNO. As hydropersulfides have been detected in biological systems at high micromolar concentrations, they could be among important biological targets of azanone. The recent study of Zarenkiewicz et al. (2021) on HNO reaction with hydropersulfides shows that RSSH are more potent traps for HNO, than RSH. They also reported that HNO reaction with small molecule RSSH leads to the formation of various RSS_n_SR and RSS-NH-S_n_R species (Zarenkiewicz et al., 2021).

#### 2.4.2 Reaction of Azanone With Aryl Sulfinates

Similar to sulfites, organic sulfinates are nucleophiles capable of HNO scavenging. The corresponding Piloty acid analogs (used as thermal HNO donors) would be expected to be the products of those reactions (Reaction 36). The rate constant for the HNO reaction of benzenesulfinate anion (C_6_H_5_SO_2_^−^), the decomposition product of Piloty’s acid, has been determined as *k*_benzenesulfinate_ = (4.4 ± 0.9) × 10^4^ M^−1^s^−1^ (Smulik-Izydorczyk et al., 2017).

$${\text{RSO}}_{2}^{-}+\text{HNO}+{\text{H}}^{+}\to {\text{RSO}}_{2}\text{NHOH}$$ (reaction 36)

The rate constants of HNO reactions with 2-bromo-, 2-chloro- and 2-trifluoromethylbenzenesulfinates (2-BrPhSO_2_^−^, 2-ClPhSO_2_^−^ and 2-CF_3_PhSO_2_^−^) were also determined and are equal to (5.0 ± 1.2) × 10^4^ M^−1^s^−1^, (3.0 ± 0.7) × 10^4^ M^−1^s^−1^ and (1.1 ± 0.2) × 10^4^ M^−1^s^−1^, respectively (Smulik-Izydorczyk et al., 2017; Smulik-Izydorczyk et al., 2019). Compared to the rate constant for the reaction of HNO with nitrite ions (the products of decomposition of Angeli’s salt, k ∼ 10^3^ M^−1^s^−1^), the rate constant for the reaction between HNO and benzenesulfinate (the products of the decomposition of Piloty’s salt) is one order of magnitude higher.

#### 2.4.3 Reaction of Azanone With *C*- and *S*-Nitrosocompounds

It has been shown that the reaction of nitrosobenzene with HNO results in the formation of stable *C*-diazeniumdiolate product, cupferron (Reaction 37) (Shoeman and Nagasawa, 1998). The second-order rate constant of that reaction was recently estimated to be higher than 1.5 × 10^5^ M^−1^s^−1^ (Smulik-Izydorczyk et al., 2017). In the same study, the reactivity of 2-nitroso-1-naphthol toward HNO was also studied, and the rate constant was determined to be equal to (1.0 ± 0.2) × 10^6^ M^−1^s^−1^ (Smulik-Izydorczyk et al., 2017).

In 1998, the study on the reaction of HNO (generated from Angeli’s salt) with *S*-nitrosoglutathione (GSNO) was reported (Wong et al., 1998). It was shown that the incubation of GSNO with Angeli’s salt results in *S*-nitrosothiol decomposition and the generation of ^•^NO. The proposed reaction mechanism included the HNO attack at the nitrogen atom of the -SNO group resulting in the formation of *S*-diazeniumdiolate, which subsequently decomposes with the generation of ^•^NO and GSH.

$$\text{RSNO }+\text{ HNO }\to \;\left[{\text{RS}-\text{NONO}}^{-}\right]{\;+\text{ H}}^{+}\;\to {\text{ RSH }+\;2\;}^{•}\text{NO}$$ (reaction 37)

The formation of *S*-diazeniumdiolate transient product has also been recently proposed for the reaction of thiols with ^•^NO, resulting in the formation of HNO (Reaction 38) (Neuman et al., 2021).

$${\text{RSH }+\;2\;}^{•}\text{NO }\to \;\left[{\text{RS}-\text{NONO}}^{-}\right]\;\to \text{ RSNO }+\text{ HNO}$$ (reaction 38)

The second-order rate constant of the reaction of HNO with GSNO has been determined to be equal to (2.4 ± 0.7) × 10^4^ M^−1^s^−1^ (Smulik-Izydorczyk et al., 2017).

#### 2.4.4 Reaction of Azanone With Phosphines

**SCHEME 4:**
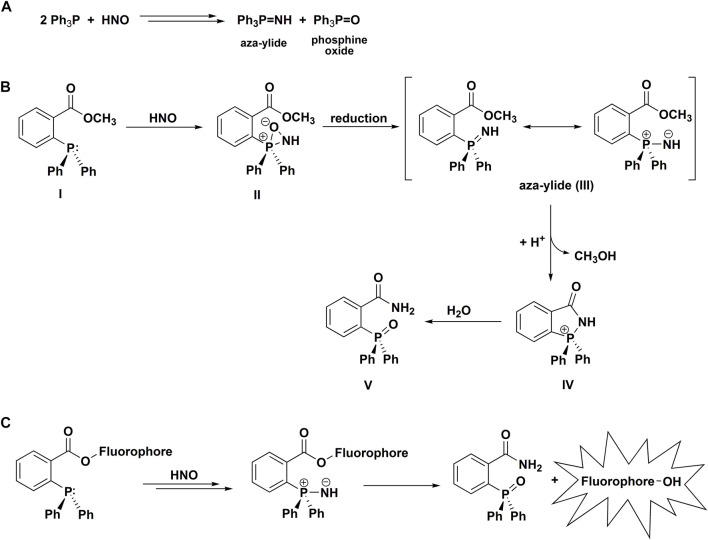
Reactivity of azanone towards phosphines **(A)**, **(B)** The Staudinger ligation of HNO-derived aza-ylide. **(C)** The mechanism of HNO detection with the use of phosphine-based fluorogenic probes.

In 2009, it was reported that HNO reacts rapidly with organic phosphines to produce corresponding phosphine oxides and aza-ylides (Scheme 4A) (Reisz et al., 2009). This report was based on the previously reported reactions of organic phosphines with *C*- and *S*-nitroso compounds resulting in the formation of phosphine oxides and aza-ylides (Haake, 1972; Wang and Xian, 2008; Zhang et al., 2009). In aqueous solutions aza-ylides hydrolyze spontaneously to yield the corresponding amine and phosphine oxide, but this unstable transient product can also be converted to a stable covalent adduct. It has been shown that an appropriately situated electrophilic trap within the phosphine structure, such as an ester functional group, can capture the nucleophilic aza-ylide in the intramolecular cyclization reaction, producing a stable amide (Scheme 4B) (Saxon and Bertozzi, 2000). Applying this strategy, Reisz et al. (2009) have shown that azanone can be effectively trapped by the methyl ester of 2-(diphenylphosphino)benzoic acid to form the corresponding amide as an HNO-specific product. Further exploration of this azanone detection strategy resulted in the development of the first phosphine-based colorimetric probe for the detection of HNO (Reisz et al., 2011). Using the competition kinetics approach and HNO reaction with GSH as a reference (*k*_GSH_ = 2 × 10^6^ M^−1^s^−1^), the rate constant for the reaction of HNO with a water-soluble phosphine, tris(2,4-dimethyl-5- sulfophenyl)phosphine trisodium salt, was estimated at 37°C and pH 7.4 to be equal to 9 × 10^5^ M^−1^s^−1^ (Reisz et al., 2011). Subsequently, Kawai et al. (2013) reported the design, synthesis and characterization of the first phosphine-based fluorogenic probe for the detection of HNO (Scheme 4C). Those seminal works were followed by a large number of reports of various arylphosphine-based fluorogenic probes for the detection of HNO, as reviewed elsewhere (Smulik-Izydorczyk et al., 2018). The detection mechanism used is based on the reaction of esters of fluorescent dyes and 2-(diphenylphosphino)benzoic acid as HNO-sensing moiety, the reaction of which with HNO leads to the release of the parent dye and fluorescence “turn on.” The reaction of HNO with a triphenylphosphine sensing site results in the formation of the aza-ylide, its subsequent intramolecular nucleophilic attack on the carbonyl carbon of the ester, leading to the liberation of the fluorescent dye and the formation of the P-oxide of the amide of 2-(diphenylphosphino)benzoic acid. The proposed mechanisms of HNO reaction with phosphines are summarized in Scheme 4.

The rate constants for the reactions of HNO with triphenylphosphine-3,3′,3″-trisulfonate and tris-carboxyethylphosphine (TCEP) were recently re-evaluated and the values determined at pH 7.4 and 25°C are equal to (3.0 ± 0.5) × 10^6^ M^−1^s^−1^ and (1.2 ± 0.3) × 10^7^ M^−1^s^−1^ (Smulik-Izydorczyk et al., 2017). The value for TCEP is in good agreement with the previously reported value of 8.4 × 10^6^ M^−1^s^−1^ (Jackson et al., 2009).

#### 2.4.5 Reaction of Azanone With C-Nucleophiles

In 2015 Guthrie et al. (2015) reported, that HNO reacts with variety of pyrazolones with the formation of corresponding HNO adducts (pyrazolone substituted hydroxylamines). They developed a general protocol for the synthesis of new and versatile class of HNO donors (hydroxylamino-pyrazolones–HAPY) from pyrazolones and Angeli’s salt, as a HNO donor. The kinetic study of HNO scavenging by pyrazolones showed that this reaction is fast and the second order rate constant at pH 7.4 and 37°C can reach value 8 × 10^5^ M^−1^s^−1^.

**FIGURE 1:**
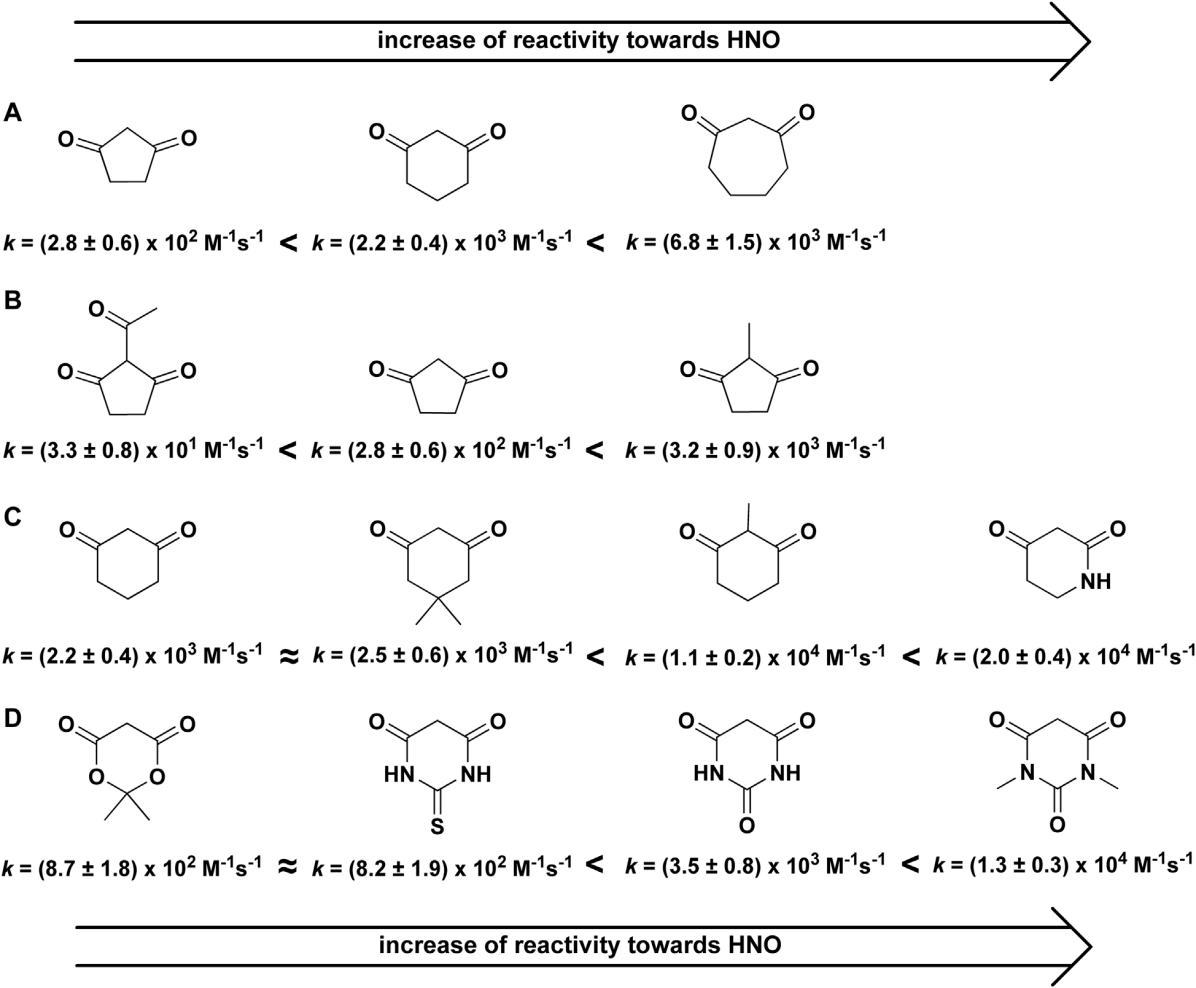
Reactivity of HNO towards cyclic *C*-nucleophiles.

Recently, it was shown that *C*-nucleophiles can act as efficient scavengers of HNO (Artelska et al., 2021). There was a strong dependence of the reactivity of *C*-nucleophiles toward HNO on nucleophile structure (Figure 1), while due to the low p*K*_a_ values all those nucleophiles are present in the solution in their deprotonated, anionic forms in aqueous solution at pH 7.4.

The reactivity of 1,3-cyclopentanedione (C_5_H_6_O_2_), 1,3-cyclohexanedione (C_6_H_8_O_2_), and 1,3-cycloheptanedione (C_7_H_10_O_2_) was studied and the obtained second order rate constants were equal to 2.8 × 10^2^ M^−1^s^−1^, 2.2 × 10^3^ M^−1^s^−1^ and 6.8 × 10^3^ M^−1^s^−1^, respectively. This indicates that the reactivity of cyclic C-nucleophiles toward HNO significantly increases with an increase in their ring size (Figure 1A). Methylation of α-carbon also resulted in a marked increase in the reactivity of *C*-nucleophiles (Figures 1B,C). The acylation of the same position has the opposite effect to methylation and results in decreased rate constant (Figure 1B).

The substitution of one or more carbon atoms in the ring of 1,3-cyclohexanedione with nitrogen resulted in an increase in *C*-nucleophile reactivity toward HNO (Figure 1C). In the case of 2,4-piperidinedione and 1-(4-methoxybenzyl)-2,4-piperidinedione the rate constants show an increase in nucleophile reactivity toward HNO, compared to 1,3-cyclohexanedione: *k*_1,3-cyclohexanedione_ = 2.2 × 10^3^ M^−1^s^−1^, *k*_2,4-piperidinedione_ = 2.0 × 10^4^ M^−1^s^−1^ and *k*_1-(4-methoxybenzyl)-2,4-piperidinedione_ = 1.4 × 10^4^ M^−1^s^−1^.

The reactivity of barbituric acid, 1,3-dimethylbarbituric acid, 2-thiobarbituric acid and Meldrum’s acid (all those cyclic C-nucleophiles contain two heteroatoms in the ring) towards azanone (Figure 1D) was also reported. Barbituric acid had similar reactivity toward HNO to 1,3-cyclohexanedione. In the case of 1,3-dimethylbarbituric acid a slight increase in the second order rate constant was observed. The reactivity of 2-thiobarbituric acid and Meldrum’s acid toward HNO was, however, rather low: *k*_2-thiobarbituric acid_ = 8.2 × 10^2^ M^−1^s^−1^ and *k*_Meldrum’s acid_ = 8.7 × 10^2^ M^−1^s^−1^.

**SCHEME 5:**
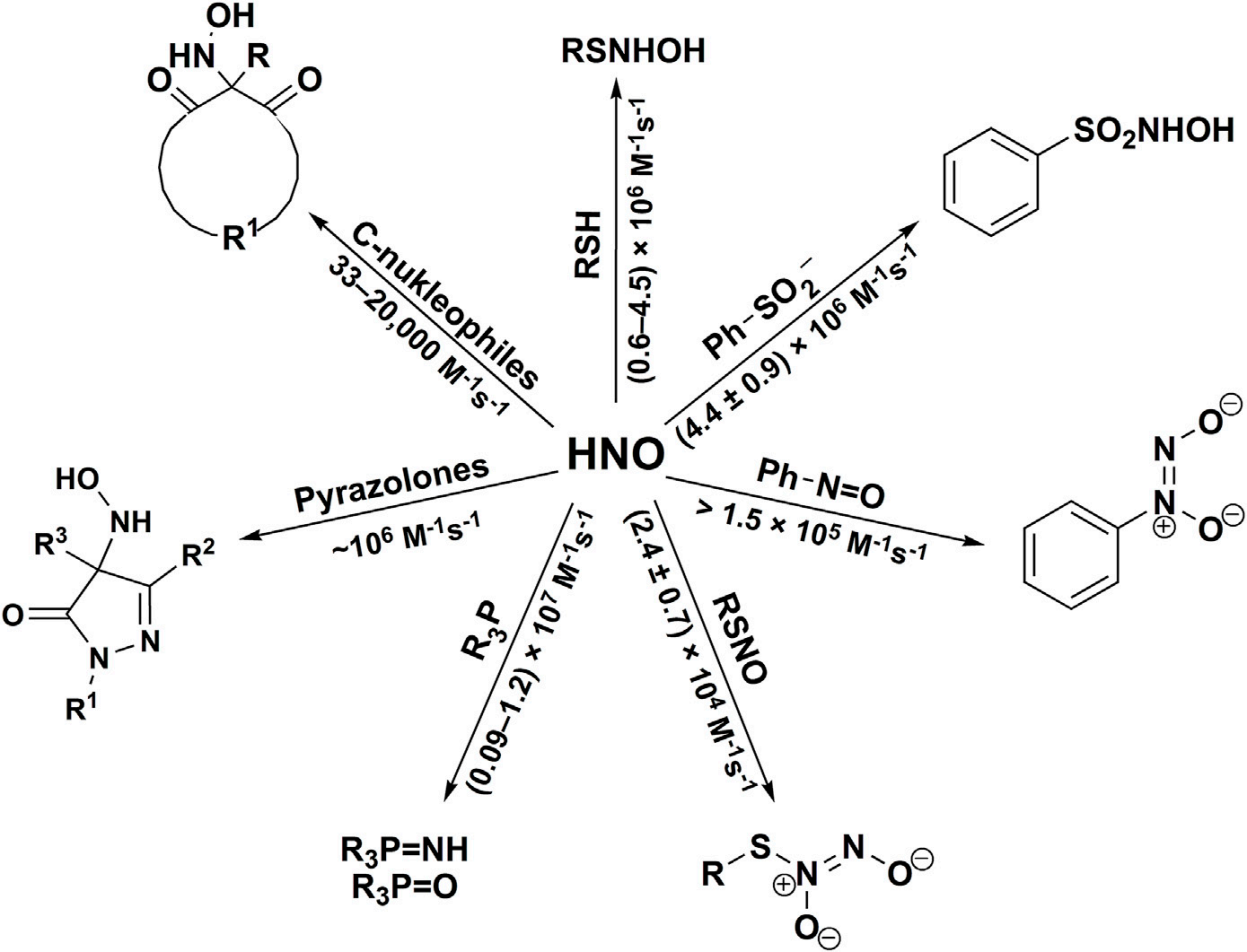
The reactivity of HNO towards organic nucleophiles.

The reactivity of HNO towards different classes of organic nucleophiles has been summarized in Scheme 5.

### 2.5 Oxidation of HNO to ^•^NO.

From the recent estimations of the one-electron reduction potential of ^•^NO (*E*°’ (^•^NO/^1^HNO) = −0.16 V for pH 7) (Venâncio et al., 2017), it could be expected that HNO can be oxidized to ^•^NO even by relatively weak one-electron oxidants. In fact, it has been shown that HNO is able to reduce cytochrome *c* (cyt *c* (Fe^3+^)) (*E*°’ = 0.256 V (Koller and Hawkridge, 1985)), Cu,Zn-SOD (*E*°’ = 0.403 V (St. Clair et al., 1992)), aminoxyl radicals: TEMPO (2,2,6,6-tetramethylpiperidine 1-oxyl, *E*°’ = 0.204 V (Kato et al., 1995)), TEMPOL (4-hydroxy-2,2,6,6-tetramethylpiperidine 1-oxyl, E°’ = 0.226 V (Kato et al., 1995)) and 3-carbamoyl-PROXYL (3-carbamoyl-2,2,5,5 -tetramethyl-1-pyrrolidineoxyl, *E*°’ = 0.102 V (Kato et al., 1995)) and nitronyl nitroxide radicals: PTIO (2-phenyl-4,4,5,5-tetramethylimidazoline-1-oxyl-3-oxide, *E*°’ = 0.270 V (Goldstein et al., 2003)) and c-PTIO (2-(4-carboxyphenyl)-4,4,5,5-tetramethylimidazoline-1-oxyl-3-oxide, *E*°’ = 0.270 V (Goldstein et al., 2003)).

In 1988 it was reported that the treatment of cyt *c*(Fe^3+^) with an excess of Angeli’s salt in phosphate buffer at pH 7.0 at 25°C leads to the formation of ferrocytochrome *c* (cyt *c*(Fe^2+^)) (Doyle et al., 1988). The initial rate of cyt *c*(Fe^3+^) reduction was nearly zero order in the concentration of cyt *c*(Fe^3+^) and first order in the concentration of HN_2_O_3_^−^, suggesting that the decomposition of Angeli’s salt was controlling the rate of the reaction, consistent with HNO acting as the reductant of cyt *c* (Fe^3+^) (Reaction 39). The reported yield of cytochrome *c* reduction at low [HN_2_O_3_^−^]_0_/[cyt *c* (Fe^3+^)]_0_ ratio was close to 50%.

$$\text{cyt }c\left({\text{Fe}}^{3+}\right)\;+\text{ HNO }\to \text{ cyt }c\left({\text{Fe}}^{2+}\right){\;+\;}^{•}{\text{NO }+\text{ H}}^{+}$$ (reaction 39)

Two close values of the second-order rate constant of cyt *c*(Fe^3+^) reduction by HNO have been reported in the literature: *k*_39_ = 2 × 10^4^ M^−1^s^−1^, (Liochev and Fridovich, 2003), and *k*_39_ = 4 × 10^4^ M^−1^s^−1^ (Miranda et al., 2003b). It is worth to note that the reduction of cyt *c*(Fe^3+^) in aqueous solutions of Angeli’s salt was not observed in the presence of O_2_ (Liochev and Fridovich, 2001) further supporting the involvement of HNO in the reduction process. Moreover, it was observed that cyt *c* (Fe^2+^) is oxidized to cyt c(Fe^3+^) in aerated solutions of Angeli’s salt (Liochev and Fridovich, 2002). Those observations can be explained, taking into account the formation of ONOO^−^ in the reaction of HNO and O_2_ (Reaction 20) and subsequent reoxidation of cyt *c*(Fe^2+^) by ONOO^−^ (Reaction 40, *k*_40_ = 1.3 × 10^4^ M^−1^s^−1^ at pH 7.4 and 25°C, (Thomson et al., 1995)) or ONOO^−^-derived one-electron oxidants, the explanation already proposed by the authors (Liochev and Fridovich, 2002).

$$\text{cyt }c\left(Fe^{2+}\right){\;+\text{ ONOO}}^{-}\;\to \text{ cyt }c\left({\text{Fe}}^{3+}\right)$$ (reaction 40)

Addition of Angeli’s salt to a buffered solution of Cu,Zn-SOD resulted in Cu^2+^ reduction to Cu^+^ in active site of the enzyme, which was attributed to the formation and oxidation of HNO (Reaction 41) (Liochev and Fridovich, 2001; 2002).

$${\text{Cu}}^{2+}\text{, Zn}-\text{SOD }+\text{ HNO }\to {\text{ Cu}}^{+}{\text{, Zn}-\text{SOD }+\;}^{•}{\text{NO }+\text{ H}}^{+}$$ (reaction 41)

Two values differing by one order of magnitude for the second-order rate constant of the HNO-dependent Cu^2+^, Zn-SOD reduction have been reported: 8 × 10^4^ M^−1^s^−1^, (Liochev and Fridovich, 2003), and *∼*1 × 10^6^ M^−1^s^−1^ (Miranda et al., 2003b). Interestingly, the occurrence of the reverse reaction, conversion of ^•^NO into HNO by the reduced form of Cu, Zn-SOD has been also proposed (Murphy and Sies, 1991). Clearly, further research on the reaction kinetics, thermodynamics and mechanism is warranted.

Other mild one-electron oxidants reported to oxidize HNO to ^•^NO are aminoxyl radicals (also known as nitroxide radicals or nitroxyl radicals). In an elegant kinetic study on the reactivity of nitroxides toward HNO with the use of EPR spectrometry, the reduction of cyclic stable aminoxyl radicals (RN-O^•^) by HNO to the corresponding hydroxylamines (RN-OH) (Reaction 42) was reported. (Samuni et al., 2013).

$${\text{RN}-\text{O}}^{•}\;+\text{ HNO }\to {\text{ RN}-\text{OH }+\;}^{•}\text{NO}$$ (reaction 42)

The second-order rate constants of HNO reactions with RN-O^•^ radicals have been determined with the use of the competition kinetics against the reference reaction of HNO with ferric myoglobin (MbFe^III^). In the presence of HNO donor, MbFe^III^ undergoes reductive nitrosylation with the formation of relatively stable ferrous nitrosyl complex, MbFe^II^NO, which can be followed by UV-Vis absorption spectroscopy. Using *k*_MbFe(III)_ = 8 × 10^5^ M^−1^s^−1^ (Miranda et al., 2003b) as a reference value, the rate constant values for HNO-derived reduction were determined for TEMPO (*k*_TEMPO_ = (1.4 ± 0.2) × 10^5^ M^−1^s^−1^), TEMPOL (*k*_TEMPOL_ = (1.4 ± 0.2) × 10^5^ M^−1^s^−1^) and 3-carbamoyl-PROXYL (*k*_3-CP_ = (4.3 ± 0.4) × 10^4^ M^−1^s^−1^) (Samuni et al., 2013). Those values were close to previously reported rate constants for TEMPOL (*k*_TEMPOL_ = 8 × 10^4^ M^−1^s^−1^) (Miranda et al., 2003b) and TEMPO (*k*_TEMPO_ = 6.3 × 10^4^ M^−1^s^−1^) (Jackson et al., 2009) and a most recent report for 4-acetamido-TEMPO (*k* = (8 ± 2) × 10^4^ M^−1^s^−1^) (Smulik-Izydorczyk et al., 2017). Nitroxides are known to efficiently quench the excited singlet states of molecules, and when they are covalently linked to a fluorophore, the intramolecular quenching of the fluorescence signal is observed (Matsuoka et al., 2016). This property, in combination with the ability of HNO to reduce the nitroxides, enabled the design of fluorogenic probes, that can be used for the detection of HNO. In 2011, a fluorogenic probe 4-((9-acridinecarbonyl)amino)-2,2,6,6-tetramethylpiperidin-1-oxyl (TEMPO-9-AC), built by covalent linking of a stable nitroxide to acridine fluorophore, for the fluorescent detection of HNO in aqueous solution was reported (Cline and Toscano, 2011). The probe reacts with HNO to produce the highly fluorescent hydroxylamine derivative, with concomitant oxidation of HNO to ^•^NO. The probe was proposed as a chemical tool to differentiate HNO from ^•^NO. The second-order rate constant for the probe reduction by HNO was reported as 8 × 10^4^ M^−1^s^−1^ (Cline and Toscano, 2011), a value confirmed in an independent study (*k*_TEMPO-9-AC_ = (9 ± 2) × 10^4^ M^−1^s^−1^) (Smulik-Izydorczyk et al., 2017). Due to the high reactivity of aminoxyl (nitroxide) radicals towards various biological reductants (e.g., ascorbate), the nitroxide-based fluorogenic probes can be used for HNO detection only in the relatively simple chemical systems in the absence of reductants capable of reducing the probe.

In case of continuous generation of HNO, it can be expected that the generated ^•^NO will react with HNO to form the N_2_O_2_^•−^ radical anion (Reaction 6), which may react with another ^•^NO to form N_2_O_3_^−^ (Reaction 7), eventually producing N_2_O and NO_2_^−^ (Reaction 8).

**SCHEME 6:**
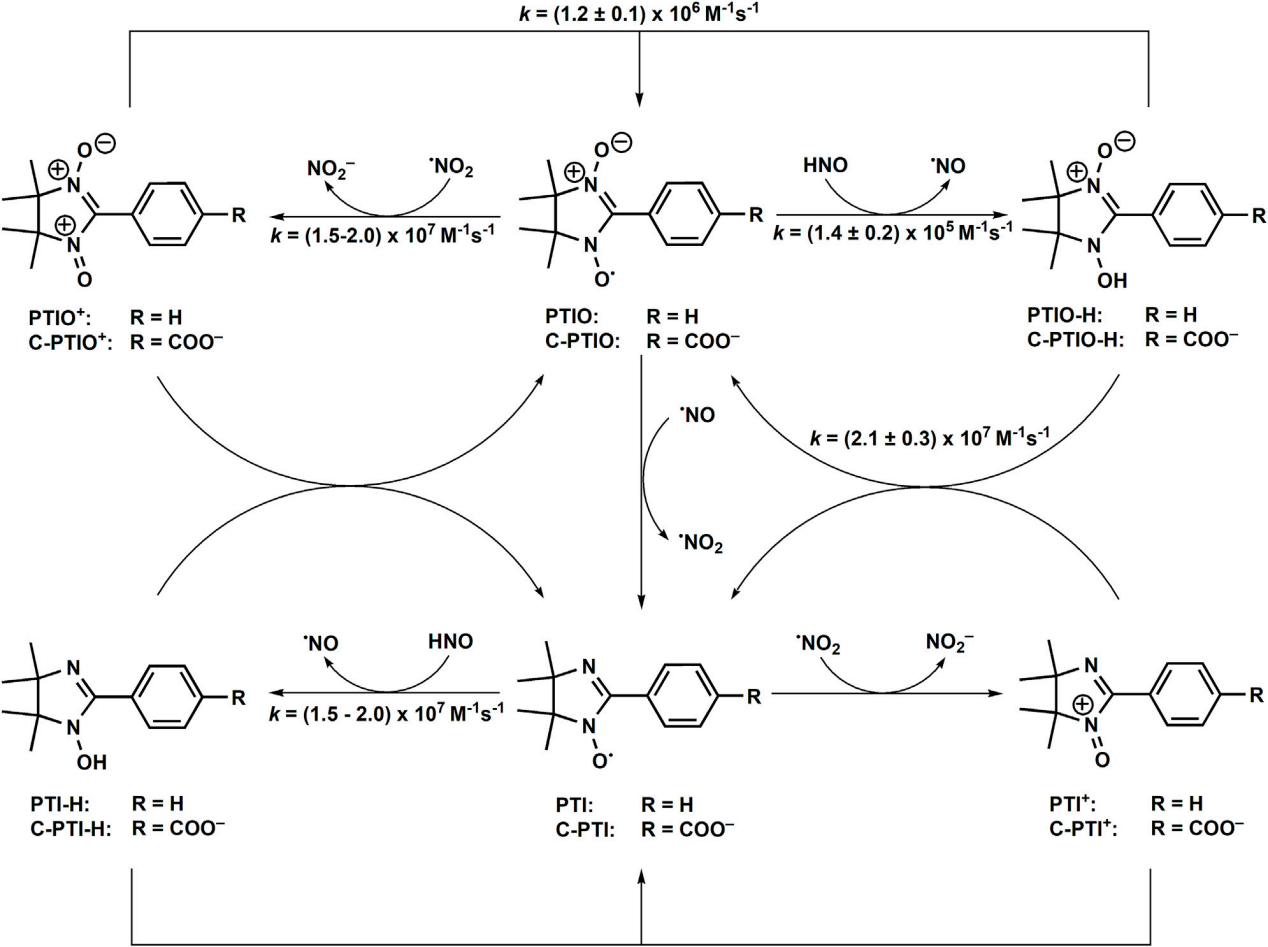
Redox reactions in HNO/nitronyl nitroxide system.

The other class of stable free radicals are nitronyl nitroxides. Those compounds, described in the scientific literature for the first time in 1968 (Osiecki and Ullman, 1968), similarly to aminoxyl radicals are sufficiently stable for isolation and characterization in the presence of O_2_. Nitronyl nitroxides, such as 2-phenyl-4,4,5,5-tetramethylimidazoline-1-oxyl 3-oxide (PTIO), are widely used in redox biology studies as scavengers of ^•^NO (Akaike et al., 1993; Joseph et al., 1993). PTIO and its analogs were shown to react with ^•^NO to form the corresponding imino nitroxides (e.g., PTI) of characteristic EPR spectrum and ^•^NO_2_ (Akaike et al., 1993; Joseph et al., 1993) (Scheme 6). The reported values of the second order rate constants of nitronyl nitroxides reaction with ^•^NO are in the range (0.4–16) × 10^4^ M^−1^s^−1^ (Akaike et al., 1993; Woldman et al., 1994; Goldstein et al., 2003; Rosen et al., 2003). In 2010 it was demonstrated that nitronyl nitroxides react with HNO to form the corresponding hydroxylamines (Scheme 6) (Samuni et al., 2010).

Due to the subsequent reactions of nitronyl nitroxide and imino nitroxide radicals with ^•^NO, resulting in the formation of highly oxidizing ^•^NO_2_ radicals, the chemistry of such a system is rather complex. The second-order rate constants of the HNO reaction with nitronyl nitroxides have been determined with the use of competition kinetics against MbFe^III^ using *k*_MbFe(III)_ = 8 × 10^5^ M^−1^s^−1^ (Miranda et al., 2003b) as a reference value. The determined rate constant values for PTIO and c-PTIO reduction by HNO at pH 7 were identical: *k*_PTIO_ = *k*_c-PTIO_ = (1.4 ± 0.2) × 10^5^ M^−1^s^−1^. Independently, the kinetics of c-PTIO reaction with HNO was determined using NH_2_OH as a competing agent and the *k*_C-PTIO_/*k*_23_ ratio of 5.5 ± 0.9 was obtained (Bobko et al., 2013). Using *k*_23_ = (4.0 ± 0.3) × 10^3^ M^−1^s^−1^ (Jackson et al., 2009) or *k*_23_ = (2.1 ± 0.4) × 10^4^ M^−1^s^−1^ (Smulik-Izydorczyk et al., 2017) one can calculate *k*_C-PTIO_ = (2.2 ± 0.5) × 10^4^ M^−1^s^−1^ (Bobko et al., 2013; Bobko and Khramtsov, 2014) or *k*_C-PTIO_ = (1.2 ± 0.4) × 10^5^ M^−1^s^−1^. It has been also shown, that ^•^NO_2_ radicals formed in such a system can oxidize Angeli’s salt anion to transient radical product, that decomposes to NO_2_^−^ and ^•^NO (Reaction 43, *k*_43_ = 7.7 × 10^6^ M^−1^s^−1^) (Bobko and Khramtsov, 2014).

N2O32-+NO2•→[N2O3•-]+NO2-→2 NO2-+N•O (reaction 43)

The kinetics of HNO reaction with nitronyl nitroxide radicals was further reevaluated by the competition kinetics approach, using hydroxylamine as an HNO scavenger and Fe(CN)_6_^4−^ anion as a ^•^NO_2_ radical scavenger (Bobko and Khramtsov, 2015). Such a system is still relatively complex as Fe(CN)_6_^4−^ and Fe(CN)_6_^3−^ anions are in redox equilibrium with nitronyl nitroxide radicals and Fe(CN)_6_^3−^ anion is an oxidant of HNO. Based on the established competition, the *k*_c-PTIO_/*k*_23_ ratio of 1.7 ± 0.2 and $k_{\text{Fe}{\left(\text{CN}\right)}_{6}^{3-}}/k_{23}$ ratio of 0.24 ± 0.17 were obtained. Using the values *k*_23_ = (4.0 ± 0.3) × 10^3^ M^−1^s^−1^ (Jackson et al., 2009) or *k*_23_ = (2.1 ± 0.4) × 10^4^ M^−1^s^−1^ (Smulik-Izydorczyk et al., 2017) one can calculate *k*_c-PTIO_ = (6.8 ± 1.3) × 10^3^ M^−1^s^−1^ and $k_{\text{Fe}{\left(\text{CN}\right)}_{6}^{3-}}$ = (9.6 ± 7.5) × 10^2^ M^−1^s^−1^ (Bobko and Khramtsov, 2015) or *k*_C-PTIO_ = (3.6 ± 1.1) × 10^4^ M^−1^s^−1^ and $k_{\text{Fe}{\left(\text{CN}\right)}_{6}^{3-}}$ = (5.0 ± 4.5) × 10^3^ M^−1^s^−1^ (Smulik-Izydorczyk et al., 2017).

### 2.6 Reactivity of HNO With Metal-Porphyrins

The reaction of HNO with heme-proteins has been studied extensively for nearly 40 years. Among the studied heme-enzymes are myoglobin (Mb), hemoglobin (Hb), horseradish peroxidase (HRP), catalase (cat), cytochrome *c* (cyt *c*), soluble guanylate cyclase (sGC), and cytochromes P450 (CYP450) (Reisz et al., 2010). The reactions between HNO and Mb as well as HNO and Hb are well documented. The oxygenated ferrous heme of myoglobin (oxymyoglobin, Mb (Fe^II^)O_2_) reacts with HNO forming ferric heme and possibly ^•^NO and H_2_O_2_ (reaction 44) (Doyle and Mahapatro, 1984; Doyle et al., 1988) and the rate constant for this reaction was determined to be *k*_44_ = 1 × 10^7^ M^−1^s^−1^ (Miranda et al., 2003b). ^•^NO formed upon HNO oxidation reacts with another Mb (Fe^II^)O_2_ molecule with rate constant *k*_45_ = 3–5 × 10^7^ M^−1^s^−1^ producing ferric heme and NO_3_^−^ (Reaction 45) (Doyle and Hoekstra, 1981; Doyle et al., 1983; Herold et al., 2001). Identical mechanism including similar reaction rate constants can be proposed for Hb(Fe^II^)O_2_ (Herold et al., 2001; Reisz et al., 2010). Overall, it can be stated that both Mb (Fe^II^)O_2_ and Hb(Fe^II^)O_2_ convert HNO to ^•^NO rapidly. It is worth to emphasize that in the literature an alternative mechanism for the heme (Fe^II^)O_2_/HNO reaction was also proposed. In that mechanism, two ferric hemes, hydroxylamine and O_2_ are the final products (Miranda, 2005).

$${\text{heme}-\text{Fe}}^{\text{II}}{\text{O}}_{2}{\;+\text{ HNO }+\text{ H}}^{+}\to {\text{ heme}-\text{Fe}}^{\text{III}}{\;+\;}^{•}{\text{NO }+\text{ H}}_{2}{\text{O}}_{2}$$ (reaction 44)

heme-FeIIO2+N•O→heme-FeIII+NO3- (reaction 45)

Deoxymyoglobin (Mb (Fe^II^)) reacts with HNO forming coordination complex Mb (Fe^II^)HNO (Reaction 46). Originally the rate constant of this reaction was estimated to be equal to 1.4 × 10^4^ M^−1^s^−1^ (Sulc et al., 2004). The re-evaluation of reaction kinetics using a singular value decomposition (SVD) method provided a corrected value of 3.7 × 10^5^ M^−1^s^−1^ (Zapata et al., 2013; Zapata et al., 2017). The reaction of Mb (Fe^II^) with HNO is followed by subsequent reaction of Mb (Fe^II^)HNO with HNO yielding Mb (Fe^III^)NO, with the rate constant estimated as 1.67 × 10^4^ M^−1^s^−1^ (reaction 47) (Zapata et al., 2013; Zapata et al., 2017). For deoxyhemoglobin the analogical mechanism can be proposed and the rate constant of HNO reaction with Hb(Fe^II^) is equal to *ca.* 2.0 × 10^5^ M^−1^s^−1^ (Kumar et al., 2009). For other ferrous globins the rate constants of the reaction with HNO are in the range 1.2–9.0 × 10^5^ M^−1^s^−1^ (Kumar et al., 2009).

$${\text{heme}-\text{Fe}}^{\text{II}}+\text{HNO}\to {\text{heme}-\text{Fe}}^{\text{II}}\text{HNO}$$ (reaction 46)

$${\text{heme}-\text{Fe}}^{\text{II}}\text{HNO}+\text{HNO}\to {\text{heme}-\text{Fe}}^{\text{III}}\text{NO}+\left[{\text{H}}_{2}{\text{NO}}^{•-}\right]$$ (reaction 47)

The ferric hemes of metmyoglobin and methemoglobin undergo reductive nitrosylation in the presence of HNO producing the ferrous-nitrosyl complexes, Mb (Fe^II^)NO and Hb(Fe^II^)NO, respectively (reaction 48) (Miranda et al., 2003b; Miranda, 2005; Reisz et al., 2010). This reaction proceeds *via* the direct Fe^III^–HNO complex formation, followed by an electron transfer (Reisz et al., 2010). The rate constant for the reductive nitrosylation of metMb by HNO was originally estimated to be 8 × 10^5^ M^−1^s^−1^ (Miranda et al., 2003b). Recent re-evaluation using SVD method led to the conclusion that the rate constant is about three times lower (*k*_48_ = 2.75 × 10^5^ M^−1^s^−1^) (Zapata et al., 2013; Zapata et al., 2017).

$${\text{heme}-\text{Fe}}^{\text{III}}+\text{HNO}\to {\text{heme}-\text{Fe}}^{\text{II}}\text{NO}+{\text{H}}^{+}$$ (reaction 48)

The rate constant of reductive nitrosylation of metHb is difficult to determine due to the side reaction of HNO with β-93 cysteines of hemoglobin (Doyle et al., 1988). Nonetheless, the observed rate of HbNO formation for the chemically modified hemoglobin containing thioacetamide derivative instead of β-93 cysteines was in agreement with the rate of MbNO formation (Doyle et al., 1988).

Catalase and HRP, similarly to metMb and metHb, are 5-coordinate ferric heme proteins and undergo reductive nitrosylation upon exposure to HNO, with estimated rate constants equal to 3 × 10^5^ M^−1^s^−1^ and 2 × 10^6^ M^−1^s^−1^ (Reaction 48) (Miranda et al., 2003b). The heme-Fe^II^NO adducts formed in case of both enzymes are stable under anaerobic conditions, but in the presence of O_2_ the ferric hemes are regenerated and NO_3_^−^ is formed (Reaction 49) (Huang et al., 2004; King, 2005).

$${\text{heme}-\text{Fe}}^{\text{II}}\text{NO}+{\text{O}}_{2}\to {\text{heme}-\text{Fe}}^{\text{III}}+{\text{NO}}_{3}^{-}$$ (reaction 49)

The reaction of cyt *c*(Fe^III^) with HNO results in the reduction of iron heme and the formation of ^•^NO, as already discussed in Section 2.5. (*Oxidation of HNO to NO*) (Liochev and Fridovich, 2003; Miranda et al., 2003b). It has been proposed that iron-nitrosyl complex is an unstable intermediate of this reaction (Doyle et al., 1988).

In the case of sGC there is no estimated rate constant for its reaction with HNO. Miller and coworkers proposed that activation of sGC proceeds directly through HNO and the ferrous heme reaction without conversion of HNO to ^•^NO (Miller et al., 2009). The expected product is sGC(Fe^II^)HNO complex (Reaction 46) but not sGC(Fe^II^)NO. sGC(Fe^III^) is unreactive toward HNO or ^•^NO. However, a contrary report was published, where the HNO-dependent activation of sGC was excluded (Zeller et al., 2009). That report suggested that the secondary reaction between sGC(Fe^II^)HNO and HNO that follows to the formation of sGC(Fe^II^)NO (reaction 47) is responsible for the activation of sGC (Zapata et al., 2013).

It was shown that the ferric heme of cytochrome P450 is reduced by HNO to heme-Fe^II^(NO) complex similarly to other heme enzymes (Reaction 48) (Miranda et al., 2003a). The interaction with HNO and the formation of the ferrous nitroxyl complex leads to the inactivation of CYP450.

On the basis of the described examples of HNO reactions with heme proteins the following conclusions can be drawn. Oxy-heme centers are the fastest scavengers of HNO, contrary to ferrous- and ferric-hemes centers, for which the values of the rate constant are at least an order of magnitude lower. The ferric forms of heme-proteins react with HNO producing appropriate ferrous heme–NO complexes with rate constants in the range between 4 × 10^4^ M^−1^s^−1^ and 2 × 10^6^ M^−1^s^−1^ (Reaction 48, Table 2). The two orders of magnitude span of the rate constants indicates that there are additional factors controlling the rate of this reaction. It has been suggested that in the case of cyt *c*, for which rate constant of reductive nitrosylation is the lowest one, the very likely reason of this phenomenon is the axially bounded histidine-18 and methionine-80 to the heme iron (Miranda, 2005). Thus, to bind to iron, HNO molecule needs to displace the axial ligand during the reaction. Another parameter that may control the kinetics of the HNO binding to the ferric center is the lability of the water ligand (Suarez et al., 2007; Alvarez et al., 2014). It is very likely that ferric hemes of proteins and ferric-porphyrins are hexacoordinated centers with axially bounded water molecules or hydroxide ions and the rate limiting step of the HNO binding is the release of such ligands (Suarez et al., 2007; Alvarez et al., 2014). For comparison, in ferrous hemes the water ligand is weakly bound, which correlates with higher rate constants of ^•^NO binding to the ferrous hemes of Hb or Mb (∼10^7^ M^−1^s^−1^) (Fukuto et al., 2013).

Based on the determined rate constants, it can be expected that heme proteins constitute one of the biological targets of HNO. Porphyrins are used as model compounds of heme-proteins that enable structure/reactivity studies with HNO without unnecessary interactions of HNO with a protein matrix. Microperoxidase-11 is an example of a pentacoordinate porphyrin-iron complex with axially bound histidine that enabled the evaluation of the influence of such ligand on the kinetics of HNO/porphyrin reaction (Suarez et al., 2007). The measured rate constant for this reaction (6.4 × 10^4^ M^−1^s^−1^) (Suarez et al., 2007) is in reasonable agreement with the rate constants of HNO reactions with other metal-porphyrins (Table 2) indicating that axial histidine does not interfere with the HNO/iron reaction.

The kinetics of HNO reaction with other porphyrins containing iron and manganese were also studied and the appropriate rate constants are compiled in Table 2 (Suarez et al., 2007; Alvarez et al., 2014). Such reactivity is important not only from the point of view of the biological relevance of such complexes as heme models, especially in the case of iron porphyrins, but also due to the potential application for detection and stabilization of HNO. The formation of nitrosyl adducts by Fe- or Mn-porphyrins is accompanied by the shift in the Soret band that can be followed by UV-Vis absorption spectroscopy (Alvarez et al., 2014). Comparing the rate constants for Fe(III)- and Mn(III)-porphyrins (Table 2) it can be seen that the reactivity of Mn(III)-porphyrins toward HNO is similar to Fe(III)-porphyrins (Alvarez et al., 2014). Taking into account the fact that in the case of Mn(III)-porphyrins the formation of nitrosyl adduct is accompanied by 60–100 nm shift of the Soret band relative to around 5 nm for Fe-porphyrins (Doctorovich et al., 2014; Vásquez et al., 2017), the use of Mn(III)-porphyrins for the detection of HNO seems to be a reasonable choice.

The reactivity studies of HNO and porphyrins revealed another interesting chemistry related to the HNO donor/porphyrin interactions. It was shown that the reaction of HNO/metal-porphyrin complexes proceeds according to two alternative mechanisms. In the first mechanism, HNO is released by the donor molecule and a free HNO molecule reacts with metal-porphyrin leading to the appropriate nitrosyl adduct (Reaction 48). In the second mechanism, the donor interacts directly with a metal center *via* the possible metal porphyrin-donor complex (Reactions 50a, 50b) (Alvarez et al., 2014; Vásquez et al., 2017). The measured rate constants of HNO donor binding to metal-porphyrins are in the range of 1 × 10^3^–1 × 10^4^ M^−1^s^−1^ (Table 2). Such direct decomposition of HNO donor was observed for the oxidizing metal-porphyrin complexes. In the case of reducing complexes, HNO must be first released by the donor, followed by reductive nitrosylation. This finding implies the possibility of a direct interaction of HNO donors with heme-proteins. In fact, the decomposition of Angeli’s salt by cytochrome P450 was reported (Shibata et al., 1997).

$${\text{MP}-\text{Fe}}^{\text{III}}+{\text{N}}_{2}{\text{O}}_{3}^{2-}\to {\text{MP}-\text{Fe}}^{\text{III}}\left({\text{N}}_{2}{\text{O}}_{3}^{2-}\right)\to {\text{MP}-\text{Fe}}^{\text{II}}\text{NO}$$ (reaction 50a)

$${\text{MP}-\text{Fe}}^{\text{III}}+{\text{CH}}_{3}{\text{PhSO}}_{2}\text{NHOH}\to {\text{MP}-\text{Fe}}^{\text{III}}\left({\text{CH}}_{3}{\text{PhSO}}_{2}\text{NHOH}\right)\to {\text{MP}-\text{Fe}}^{\text{II}}\text{NO}$$ (reaction 50b)

The reactivity of cobalt-complexes based on porphyrins, corroles, and corrins toward HNO was also reported (Vásquez et al., 2017; Gallego et al., 2021). Cobalamin (vitamin B_12_, Cbl (III)) is an important example, since it occurs naturally and is an essential mammalian coenzyme. It was shown that aquacobalamin (H_2_O-Cbl^+^) and hydroxycobalamin (HO-Cbl) in the presence of Angeli’s salt are converted to the nitroxyl cobalamin (NO^−^-Co^III^Cbl) (Subedi et al., 2014). The mechanism of the reaction was dependent on the pH. At high pH over 10.8 the rate determining step was the spontaneous decomposition of Angeli’s salt to HNO and nitrite, followed by the rapid reaction of HNO with the complex. At lower pH (≤ 9.9) a direct reaction between Angeli’s salt anion and H_2_O-Cbl^+^ occurs and the complex was observed. The rate constant for the AS/H_2_O-Cbl^+^ reaction at acidic pH was determined to be 122.6 ± 5.3 M^−1^s^−1^ (Subedi et al., 2014). Recent study on the reaction between H_2_O-Cbl^+^ and Piloty’s acid (PA) has shown that PA also reacts directly with H_2_O-Cbl^+^ at neutral pH (28.4 ± 0.4 M^−1^s^−1^) (Gallego et al., 2021). In turn, in basic solutions, it was shown that HO-Cbl reacts with HNO released from PA, but the rate constant of this reaction remains unknown (Gallego et al., 2021). The mechanistic and kinetic studies on the reaction of reduced cobalamins, cob (II)alamin (Cbl (II)) and cob(I)alamin (Cbl(I)) were also performed (Subedi and Brasch, 2015; 2016). The reduced derivatives of cobalamins react with HNO as well, but mechanisms of these reactions are complex. Nonetheless, the final observed product for Cbl (II) and Cbl(I) is (NO^−^-Co^III^Cbl) (Subedi and Brasch, 2015; 2016).

To summarize, the reaction scheme of HNO with heme-proteins is very complex and still remains an area that requires further studies and analyses. In addition, an interesting aspect relates to the direct reactions of HNO-donors with metal-porphyrins, of potential pharmacological relevance.

### 2.7 Reactivity of HNO Towards NADH, NADPH, Ascorbate, Indoles and Other Reductants

#### 2.7.1 HNO-Dependent NADH, and NADPH, Oxidation

The reactivity of HNO towards NADH and NADPH was a subject of several studies. The mechanism of HNO-dependent NADH and NADPH oxidation, however, is still not completely understood (Wink et al., 1998; Liochev and Fridovich, 2001; Reif et al., 2001; Kirsch and de Groot, 2002; Liochev and Fridovich, 2002; Jackson et al., 2009). In 1998, it was shown that NADPH is oxidized in aerated and deaerated Angeli’s salt solutions (Wink et al., 1998). Aerobic oxidation of NADPH was partially inhibited by TEMPOL and hydroxylamine. At low concentrations of Angeli’s salt in aerated solutions, 1 mol of NADPH was oxidized per about 4 mol of HN_2_O_3_^−^, however the exact stoichiometry of this reaction with respect to HNO is unclear (Reif et al., 2001). NADPH oxidation in such solutions was inhibitable by Cu,Zn-SOD. In an independent study, it was shown that NADPH consumption accounted for ∼50% of decomposed Angeli’s salt (Liochev and Fridovich, 2001). Both Cu,Zn-SOD and Mn-SOD were able to inhibit the oxidation of NADPH by HNO. When Angeli’s salt was aerobically oxidizing NADPH in the presence of cyt *c*(Fe^3+^) (10–40 μM), the Cu,Zn-SOD-inhibitable reduction of cyt *c*(Fe^3+^) was observed. The Cu,Zn-SOD concentration was selected to be sufficient to compete with cyt *c*(Fe^3+^) for O_2_^•–^, but too low to detectably inhibit NADPH oxidation. That experiment strongly suggested the generation of O_2_^•–^ in HNO/O_2_/NADPH system. The authors proposed that NADPH is oxidized in such a solution to NADP^•^ radicals, which subsequently reduce O_2_ to O_2_^•−^ with the formation of NADP^+^. At higher concentration (∼2 μM), Cu,Zn-SOD inhibited by ∼50% the oxidation of NADPH (Liochev and Fridovich, 2001). Similar effect of Cu,Zn-SOD on the NADH oxidation in aerated Angeli’s salt solution was reported subsequently (Kirsch and de Groot, 2002). In that paper it was also demonstrated that NADH oxidation in Angeli’s salt solution can be significantly inhibited by the addition of a ^•^NO donor DEA-NONOate, when [^•^NO]/[HNO] ratio is closed to 2. Under those conditions, HNO reaction with ^•^NO leading to the formation of N_2_O_2_^•−^ and its subsequent reactions with ^•^NO were favored (Reactions 6, 7). The second-order rate constant of the reactions between NADH and HNO equal to *k*_51_ = (1.1 ± 0.2) × 10^4^ M^−1^s^−1^ was reported (Jackson et al., 2009). Similar value *k*_52_ = (1.3 ± 0.4) × 10^4^ M^−1^s^−1^ was also determined for NADPH. In the direct reaction of HNO with NADH or NADPH in deaerated Angeli’s salt solution, one may expect the formation of hydroxylamine as an HNO reduction product (Reactions 51, 52). However, experimental data did not support that hypothesis (Jackson et al., 2009). Clearly, further research on this type of HNO reactions is needed.

$$\text{NADH}\;+\;\text{HNO}\;\to \;{\text{NAD}}^{+}\;\left(+\;{\text{NH}}_{2}\text{OH}\right)$$ (reaction 51)

$$\text{NADPH}\;+\text{ HNO}\;\to \;{\text{NADP}}^{+}\;\left({+\text{NH}}_{2}\text{OH}\right)$$ (reaction 52)

#### 2.7.2 Ascorbate, Trolox and Selenomethionine

Using the competition kinetic approach and HNO reaction with NADH as a reference, the rate constants for the reaction of HNO with ascorbate, trolox and selenomethionine were determined to be equal to 1.1 × 10^5^, 2 × 10^4^ and 9 × 10^3^ M^−1^s^−1^, respectively (Jackson et al., 2009). The mechanisms of those reactions remain to be established.

#### 2.7.3 Indoles

The results of the studies on the reaction of various indolic compounds (e.g., *N*-acetyl-L-tryptophan, indol-3-acetic acid and melatonin) with Angeli’s salt in aerobic buffered aqueous solutions (pH 7.4) has been presented in several scientific reports (Suzuki et al., 2004; Peyrot and Ducrocq, 2007; Keceli et al., 2014). All these studies show that this reaction leads to the formation of *N*-nitroso derivative as the main product. The yield of its formation is rather low (below 20%) and depends on the indolic compound: Angeli’s salt concentrations ratio (Peyrot et al., 2006; Keceli et al., 2014). It was shown that the yield of *N*-nitroso derivative is significantly higher in the presence of bicarbonate (Suzuki et al., 2004), which may suggest the participation of peroxynitrite in the *N*-nitrosation mechanism. The recent study of Keceli et al. (2014) on the reaction of HNO with *N*-acetyl-L-tryptophan and small peptides containing either tryptophan or both tryptophan and cysteine residues, has shown that in the presence of cysteine, excess HNO is required for efficient TrpNO formation, clearly indicating, that tryptophan residues are significantly less reactive towards HNO, than cysteine residues.

## 3 Conclusion

HNO show a different chemical reactivity than ^•^NO, and may be expected to exhibit unique physiological activity. The experimental limitations due to its instability under physiological conditions provide a significant hindrance in study design to further explore HNO chemistry. The development of new donors and rapid generation methods for time-resolved kinetic studies may overcome some of those obstacles. In addition, the combination of the experimental data with theoretical calculations may help with drawing reliable mechanistic conclusions. Finally, as a note of caution, it has to be emphasized that many rate constants of HNO reactions listed in this review were determined by competition kinetics, *via* kinetic simulations or other indirect approaches, and are based on very few values determined directly. Therefore, it can be expected that with the development of new experimental methods, the rate constants may need to be re-evaluated and the mechanistic conclusions adjusted.
